# Identification of Tissue-Specific Gene Clusters Induced by DNA Demethylation in Lung Adenocarcinoma: More Than Germline Genes

**DOI:** 10.3390/cancers14041007

**Published:** 2022-02-16

**Authors:** Anna Diacofotaki, Axelle Loriot, Charles De Smet

**Affiliations:** 1 Group of Genetics and Epigenetics, de Duve Institute, Université Catholique de Louvain, 1200 Brussels, Belgium; anna.diacofotaki@uclouvain.be (A.D.); axelle.loriot@uclouvain.be (A.L.); 2 Group of Computational Biology and Bioinformatics, de Duve Institute, Université Catholique de Louvain, 1200 Brussels, Belgium

**Keywords:** epigenetics, DNA methylation, DNA hypomethylation, cancer-germline genes, lung adenocarcinoma

## Abstract

**Simple Summary:**

Loss of DNA methylation is often observed in human tumors, but how this epigenetic alteration impacts the transcriptome of cancer cells remains largely undefined. So far, DNA hypomethylation in tumors has been associated with aberrant activation of a germline-specific gene expression program. Here, we exploited transcriptomic and methylomic datasets of lung adenocarcinoma to investigate the possibility that other gene expression programs also become ectopically activated in hypomethylated tumors. Remarkably, we found that DNA hypomethylation in lung adenocarcinoma is associated with ectopic activation of not only germline-specific genes, but also gene clusters displaying specific expression in the gastrointestinal tract, or in stratified epithelia. Interestingly, expression of genes in this latter group was of prognostic value. Together, our study brings novel insight into the transcriptomic changes associated with DNA hypomethylation in tumors, and is an incentive to explore the value of hypomethylated DNA sequences as cancer biomarkers.

**Abstract:**

Genome-wide loss of DNA methylation is commonly observed in human cancers, but its impact on the tumor transcriptome remains ill-defined. Previous studies demonstrated that this epigenetic alteration causes aberrant activation of a germline-specific gene expression program. Here, we examined if DNA hypomethylation in tumors also leads to de-repression of gene clusters with other tissue specificities. To this end, we explored transcriptomic and methylomic datasets from human lung adenocarcinoma (LUAD) cell lines, normal lung, and lung alveolar type II cells, considered as the origin of LUAD. Interestingly, DNA demethylation in LUAD cell lines was associated with activation of not only germline-specific (CG) genes, but also gene clusters displaying specific expression in the gastrointestinal tract (GI), or in stratified epithelia (SE). Consistently, genes from all three clusters showed highly specific patterns of promoter methylation among normal tissues and cell types, and were generally sensitive to induction by a DNA demethylating agent. Analysis of TCGA datasets confirmed that demethylation and activation of CG, GI and SE genes also occurs in vivo in LUAD tumor tissues, in association with global genome hypomethylation. For genes of the GI cluster, we demonstrated that HNF4A is a necessary factor for transcriptional activation following promoter demethylation. Interestingly, expression of several SE genes, in particular *FAM83A*, correlated with both tumor grade and reduced patient survival. Together, our study uncovers novel cell-type specific gene clusters that become aberrantly activated in LUAD tumors in association with genome-wide hypomethylation.

## 1. Introduction

Cell type-specific patterns of gene expression are in part determined by epigenetic mechanisms, involving chemical modifications of DNA and associated histones, which modulate the accessibility of transcription factors to the chromatin [1,2]. Once installed, epigenetic modifications are maintained through cell divisions, and exert thereby durable effects. Methylation of CpG dinucleotides in the DNA, for instance, has been associated with a closed chromatin structure, and hence long-term repression of gene transcription [1]. It was therefore proposed that DNA methylation contributes to the establishment of specific gene expression programs during differentiation of the various cell lineages [3].

It is now clear that cancer development is driven in part by epigenetic dysregulations, which cause changes in gene expression patterns [4]. Loosening of gene regulatory processes is thought to confer increased plasticity to cancer cells, thereby accelerating their progression towards a state of enhanced malignancy [5].

Global loss of DNA methylation marks was the first epigenetic alteration to be described in human tumors [6,7]. Intriguingly, DNA hypomethylation in tumors has been merely associated with ectopic activation of a cluster of germline-specific genes, including more than 100 testis-specific genes being aberrantly expressed in a wide variety of tumor types [8]. Studies confirmed that such “cancer-testis” (CT) or “cancer-germline” (CG) genes rely primarily on DNA methylation for repression in non-expressing cells, and that genome demethylation is a sufficient trigger for their concerted activation in most tumors [9,10,11,12]. Due to their highly restricted pattern of expression, CG genes were exploited as biomarkers in cancer detection, and as antigenic determinants in anti-cancer vaccinations [13]. Moreover, evidence has accumulated indicating that some CG genes contribute to tumor development, notably by regulating processes of cell proliferation, death resistance, metabolic adaptation, and DNA repair [14,15].

While DNA hypomethylation in tumors is evidently associated with aberrant activation of a large group of germline-specific genes, it is less clear if it can also lead to de-repression of gene clusters that normally show restricted expression in somatic tissues. Several examples of genes with specific expression in somatic tissues were shown to become activated in tumors in association with promoter demethylation [16,17]. However, the few genes identified could not be grouped into tissue-specific clusters, and their activation in tumors appeared less stringently associated with DNA demethylation, as compared with CG genes [18]. In fact, the very notion that DNA methylation can serve as a primary mechanism of regulation for genes with specific expression in somatic tissues remains a matter of debate [19].

In the present study, we decided to reinvestigate the possibility that DNA hypomethylation in tumors might be associated with ectopic activation of not only a germline-specific expression program, but also of somatic expression programs. To this end, we screened publicly available transcriptomic and methylomic datasets derived from human lung adenocarcinoma (LUAD) cell lines, normal lung tissues, and alveolar type II (AT2) cells, the cells at the origin of adenocarcinoma of the lung [20]. Computational analyses were performed on these datasets to identify transcripts showing coincident transcriptional de-repression and promoter demethylation in cancer cells. Of note, compared with other studies, our screening procedure did not take into account up-regulated transcripts, but focused on transcripts that are initially absent in normal cells and become induced de novo in tumor cells. The tissue specificity of the isolated transcripts was then determined by analyzing transcriptomic and methylomic databases of normal human tissues and cells. Interestingly, besides germline-specific genes, we identified other clusters of tissue-specific genes, and confirmed their activation in vivo in hypomethylated LUAD tumor tissues. The role of DNA methylation and transcription factors in regulating such genes was evaluated experimentally. Finally, we investigated if expression of the genes we identified was associated with tumor grade and patient survival in LUAD.

## 2. Materials and Methods

### 2.1. LUAD Cell Lines Datasets

For expression and methylation analyses in the 26 LUAD cell lines, FastQ files of bulk RNA-seq and methyl-seq were obtained from Suzuki and colleagues [21]. RNA-seq FastQ files were processed as previously described [22], to obtain expression levels of individual referenced and unreferenced transcripts in each cell line. For methylation analysis, read quality control and trimming of low-quality reads were performed using Trim Galore! software v0.5.0 (https://github.com/FelixKrueger/TrimGalore) (accessed on 10 February 2022). Read alignment and methylation calling were done using Bismark v0.20.0 [23]. LUAD cell lines were considered positive for the expression of a gene when the TPM was ≥2, and negative when it was <1. All accession numbers are listed in the Table S1.

### 2.2. Procedure for the Identification of DDIC and Non-DDIC Transcripts in LUAD Cell Lines

(1) *Merging of transcripts originating from the same promoter region in LUAD cell lines.* Transcripts arising from a transcriptional start site (TSS) located less than 50bp apart were considered to originate from the same promoter region. Their expression levels were therefore summed for transcript quantification. For each pooled transcript group, the TSS located at the closest 5′ end was chosen as reference.
(2) *Selection of repressed transcripts in normal lung.* Transcripts that were initially silent in AT2 cells (RNA-seq data, [24]) and normal lung (RNA-seq, Roadmap Epigenomics [25]), and exhibited a methylation level of their promoter region ≥60% in normal lung (WGBS, Roadmap Epigenomics) and AT2 cells (WGBS, [26]) were retained. Transcript repression was defined as TPM <1 and/or lack of RNA-seq reads mapping to the genomic region of each transcript. The level of methylation of the promoter region was computed by averaging the methylation values of each CpG located at TSS −/+ 400 bp for each transcript. Only genomic regions harboring at least 3 CpGs were considered for this analysis, and when coverage information was available, CpGs covered by at least 2 reads were analyzed.
(3) *Selection of activated transcripts in LUAD cell lines.* Among the previous transcript selection, we selected those that fulfilled the following criteria: first, transcripts were repressed in at least 2/26 cell lines (expression quantile 10 ≤ 0.1 TPM) and activated in at least 1/26 cell line with their maximum expression ≥2 TPM. Secondly, the minimum value of the level of methylation of their promoter region was below 40% and the maximum above 60%. These transcripts were considered as being repressed in normal lung tissue and ectopically expressed in LUAD cell lines.
(4) *Correlation between transcriptional activation and methylation of promoter region.* We computed a Pearson correlation of the expression and promoter methylation status of each transcript in LUAD cell lines. At least 7/26 LUAD cell lines had to have both transcript expression and promoter methylation information available for a correlation to be computed. We selected transcripts that showed a significant inverse correlation between their transcriptional activation and promoter methylation (*r* ≤ −0.4, *p*-value < 0.05).
(5) *Manual curation.* We visualized BAM files of the LUAD cell lines using IGV (Integrative Genomics Viewer, [27]) to determine the accuracy of the chosen TSS, confirm the transcriptional activation status, and determine transcript backbone for each of the selected transcripts. Finally, we also verified the correlation between expression and promoter methylation of each transcript by generating heatmaps (ComplexHeatmap R package v2.8.0) depicting the methylation state of each CpG located at TSS −/+ 400 bp. Transcripts that passed all the above filters were referred to as DNA-Demethylation-associated Induction in Cancer transcripts (DDIC transcripts). Non-DDIC transcripts were selected using the same procedure with different criteria for the following parameters: transcripts were originating from a gene giving rise only to that transcript (i.e., not alternative gene promoters), the corresponding gene is repressed in normal lung (lung tissue samples of GTEx show a median TPM < 0.5 and the one from Roadmap Epigenomics <1 TPM) and repressed in AT2 cells (TPM < 1). They showed no correlation between transcriptional activation and promoter demethylation (−0.1 ≤ *r* ≤ 0.1). No manual curation step was performed for non-DDIC transcripts.

### 2.3. RNA-Seq Public Datasets

(1) *Bulk RNA-seq.* Normalized expression data of samples coming from 50 normal tissues were obtained from GTEx portal (v8, [28]). Both gene expression of individual samples per tissue and median expression of all samples per tissue were downloaded. FastQ files of the following cells and tissues were downloaded from Sequence Read Archive (SRA): AT2, lung, testis, sigmoid colon, small intestine, stomach, pancreas, liver, esophagus, skin, adipose, cerebral cortex, heart and thyroid. Files were processed as previously described [29]. All accession numbers are listed in Table S3.
(2) *Single-cell RNA-seq*. Normalized expression data of skin single-cell study (scRNA-seq, [30]) were obtained from the Human Protein Atlas (HPA) v20.1 [31]. All accession numbers are listed in Table S3.

### 2.4. RNA-Seq of Cell Lines after DNA Demethylation Treatment

FastQ files of MCF7 breast cancer cell line, HMLER immortalized mammary epithelial cells, and TS603 glioma cell line, treated or not with a demethylating agent, were downloaded from SRA [32,33,34]. Read quality control was performed using FastQC software (v0.11.8) and low-quality reads were discarded using Trimmomatic software v0.38 [35]. Reads were aligned onto hg38 genome using HISAT2 v2.1.0 [36], and gene quantification was carried out with featureCounts software from the subread package v2.0.0 [37]. Expression data are depicted in TPM. For differential expression analysis, gene expression counts generated by featureCounts of each cell line were used as input for DESeq2 (v1.32.0) R package [38]. Genes with an adjusted *p*-value < 0.05 were considered differentially expressed between conditions. All accession numbers are listed in Table S4.

### 2.5. DNA Methylation Public Datasets

(1) *Normal tissues.* For cerebral cortex samples, FastQ files of whole genome bisulfite-seq (WGBS) were downloaded from SRA. Trim Galore! software v0.5.0 was used to read quality control and trimming of low-quality reads. Read alignment and methylation calling were performed using Bismark v0.20.0. For liver sample, BAM file of WGBS was downloaded from ENCODE consortium database [39], and methylation calling was performed using Bismark v0.20.0. For the rest of the normal tissues, normalized hg38 WGBS data were obtained from the ENCODE consortium database.
(2) *Primary cells.* For keratinocytes and sperm cells, WGBS hg19 normalized data were obtained from SRA [25]. Data were converted to hg38 coordinates using liftOver v1.10.0 R package. Normalized hg38 WGBS data of HUES64 were obtained from the ENCODE consortium database. For AT2 cells, FastQ files of WGBS were downloaded from SRA [26]. Trim Galore! software v0.5.0 was used to read quality control and trimming of low-quality reads. Read alignment onto hg38 genome and methylation calling were performed using Bismark v0.20.0. For all the above studies, when available, methylation information for the same CpG sequenced in forward and reverse strand were averaged. For duodenal crypt cell samples, normalized Infinium HumanMethylation 450 assays and EPICarrays data were obtained [40]. All accession numbers are listed in Table S3.

### 2.6. Immunohistochemical Data of Normal Tissues

Immunohistochemical images of the esophagus, skin and vagina were obtained from the HPA. Antibodies for the detection of A2ML1 and SERPINB5 proteins were selected based on the fact that immunohistochemical staining patterns mirrored the gene expression profile as established by RNA-seq studies of GTEx and HPA consortia. Antibodies and tissue codes are listed in Table S5.

### 2.7. Cell Culture

LXF289 lung adenocarcinoma cell line was purchased from CLS Standard and cultured in RPMI medium (CLS Standard, Waltham, MA, USA). SKMEL23 melanoma cell line, and LB996RCC renal carcinoma cell line were obtained from the Brussels branch of the Ludwig Institute Cancer Research, and were cultured as previously described [9,10]. HEK293 human embryonic kidney cells were purchased from Thermo Fischer and cultured in DMEM (Life Technologies, Carlsbad, CA, USA). HFF2 foreskin fibroblasts were kindly provided by Dr Decottignies (de Duve Institute, UCLouvain) and cultured in DMEM. All media were supplemented with 10% Fetal Bovine Serum (Sigma, Burlington, MA, USA) and 1% penicillin/streptomycin (Life Technologies).

### 2.8. Cell Treatment with 5-azadC and siRNAs

For 5-azadC treatments, tumor and normal cell lines were grown to 60–70% confluency and then treated with a single dose of 2 µM of 5-aza-2′-deoxycytidine diluted in 1:1 acetic acid/water. Cells were maintained in culture for 6 days and then harvested for RNA extraction. For combined treatment with 5-azadC and siRNAs, LXF289 cells were seeded at 200,000 cells/well of a 6w plate and were reverse transfected with siRNAs directed against HNF4A (siHNF4A), or control siRNAs directed against luciferase (siLuc, [41]) at a final concentration of 100 nM, using Lipofectamine 2000 (Invitrogen, Waltham, MA, USA). The following day, cells were treated with either 2 µM 5-azadC or 1:1 acetic acid/water as a control. Cells were harvested at day 3 and day 5 post-transfection for RNA and protein analysis. siRNA sequences are listed in Table S6.

### 2.9. RT-PCR and qPCR Analyses

RNA of normal tissue samples (lung, testis, esophagus and colon) was purchased from Ambion (Life Technologies). RNA of normal and tumor cell lines was extracted using TriPure isolation reagent (Roche, Basel, Switzerland). Reverse transcription was performed using MML-V reverse transcriptase kit (Invitrogen), random hexamers (Invitrogen), Ribolock RNAse inhibitor (Invitrogen, 20U) and 2 µg of total RNA per reaction in a final volume of 20 µL. PCR analyses were carried out using DreamTaq kit (ThermoFischer Scientific, Waltham, MA, USA) with 1/40 of the reverse transcription solution engaged per reaction in a total volume of 20 µL. qPCR analyses were performed using KAPA SYBR FAST kit (Sigma-Aldrich) with 1/40 of the reverse transcription solution engaged per reaction in a final volume of 10 µL. All reactions were carried out according to the manufacturer’s instructions. Primer sequences and experimental conditions are listed in Table S7. Different positive control cDNAs were used depending on the gene: testis (*NAP1L1*, *NAA11*, *MAGEA1*, *ACTB*), colon (*EPS8L3*, *VIL1*), esophagus (*GJB5*, *SERPINB5*, *PGLYRP3*).

### 2.10. Western Blot

At day 3 post-transfection LXF289 cells were harvested for protein extraction. Cells were lysed and proteins recovered using RIPA buffer (150 mM NaCl, 1% TritonX-100, 0.5% sodium deoxycholate, 0.1% SDS and 50 mM Tris pH8) supplemented with 1× cOmplete Mini protease inhibitor cocktail (Roche) and 1× PhosSTOP phosphatase inhibitor cocktail (Roche). 30 µg of proteins were loaded onto an 8% SDS-PAGE gel and subsequently transferred for 2 h, 240 mA onto a PVDF membrane (Millipore, Burlington, MA, USA). The membrane was blocked with 5% milk-TBS-Tween 0.1%, 1 h at RT and then incubated overnight with anti-HNF4a antibody (1:1000 CST3113; diluted in 5% milk-TBS-Tween 0.1%). The membrane was then washed 3× in TBS-Tween 0.1% and incubated at RT 1 h with a goat anti-rabbit antibody conjugated to HRP (1:10,000 Enzo Life Sciences, Farmingdale, NY, USA, ADI-SAB-300J, diluted in TBS-Tween 0.1%). The membrane was washed 3× in TBS-Tween 0.1% and revealed using SuperSignal West Pico PLUS Chemiluminescent substrate (ThermoFisher) and CL-Xposure films (Life Technologies). For Vinculin reveal, membrane was stripped with 4× NaOH 0.4M solution, blocked 1 h at RT with 5% milk-TBS-Tween 0.1%, and incubated for 1 h with anti-VCL antibody (1:100,000, Millipore 05386). A goat anti-mouse secondary antibody conjugated to HRP (1:10,000 ab205719 diluted in TBS-Tween 0.1%) was used to reveal the membrane as described above.

### 2.11. The Cancer Genome Atlas Consortium Datasets and Analyses

Bulk RNA-seq, hg19 Infinium HumanMethylation450 assay of LUAD tumor samples, and LUAD patients’ vital status were obtained through TCGAbiolinks R package v2.14.1 [42]. A list of pathology grades of TCGA-LUAD tumor samples data was compiled and kindly provided by Drs Yu and Snyder (Stanford University, Stanford, CA, USA, [43]).

(1) *Expression analysis*. FPKM expression data were converted to TPM by dividing each FPKM gene expression in a sample by the sum of all gene expressions for that sample (×10^6^). LUAD tumor samples were considered positive for the expression of a gene if they exhibited a TPM ≥ 2. They were considered negative when they showed a TPM <1. To define activating and non-activating LUAD tumor groups for each DDIC, expression quantile 20 and 80 of each gene were used as activation cut-offs (i.e., tumor samples that show a DDIC expression TPM <q20 were considered as repressing, and conversely, when TPM >q80 were considered as activating). When q80 value was <1 TPM, then all tumor samples showing a gene expression ≥1 TPM were considered positive.
(2) *Tumor grade analysis.* Tumor grades were compared in the activating and non-activating tumor groups using a Chi-squared test. Comparison was computed if there were at least 15 tumors in each group. LUAD samples qualified as grades 1.5 and 2.5 were categorized as grade 1 and grade 2, respectively.
(3) *Survival analysis.* Patient median survival time were compared between the activating and non-activating LUAD tumor groups using a Log rank test from the survival R package v3.2-11. At least 15 tumor samples had to constitute each group, to compare survival time between patients. When median survival time was not reached for a tumor group, the length of the TCGA-LUAD survival study was taken as the median survival time.
(4) *Methylation analysis*. Infinium CG probes in hg19 coordinates were converted to hg38 coordinates using liftOver v1.10.0 R package. For correlation analyses between gene expression and promoter DNA methylation, LUAD tumor samples that exhibited information for both expression and promoter methylation were analyzed. CG probes located at −/+ 400 bp of TSS of each gene were averaged to define the level of methylation of the promoter region. Pearson correlation was computed for each gene.
(5) *Global DNA methylation analysis*. Autosomal probes that showed an average of methylation higher than 0.7 in all normal lung samples (*n* = 32) were selected as a proxy for the assessment of global methylation levels in each tissue sample (*n* = 137,954 probes). To assess promoter methylation status in regard to global methylation levels, we defined two tumor subgroups based on the promoter methylation values of each DDIC in all LUAD tumor samples. We considered a first group of tumor samples that showed a methylated promoter region of each gene (i.e., the promoter methylation level was ≥quantile 80 of methylation values for that gene) and a second group that showed a demethylated promoter of each gene (promoter methylation ≤ quantile 20). Then, the global methylation level was compared between these two tumor groups using a Student’s *t*-test.

### 2.12. Statistical Analysis and Graphical Representations

Statistical analysis was computed in R v4.1.0 (http://www.R-project.org) (accessed on 10 February 2022) or GraphPad Prism (v5.0). All *p*-values were adjusted using the Benjamini-Hochberg method. For clustering of DDIC expression in normal tissues of GTEx and LUAD samples, Ward’s method with Euclidean distance were used. DDIC genes were clustered inside their tissue specific categories using the same parameters. Clustering results are represented using the ComplexHeatmap R package (v2.8.0).

## 3. Results

### 3.1. Search for Transcripts Showing DNA Demethylation-Associated Induction in LUAD Cell Lines

**Figure 1:**
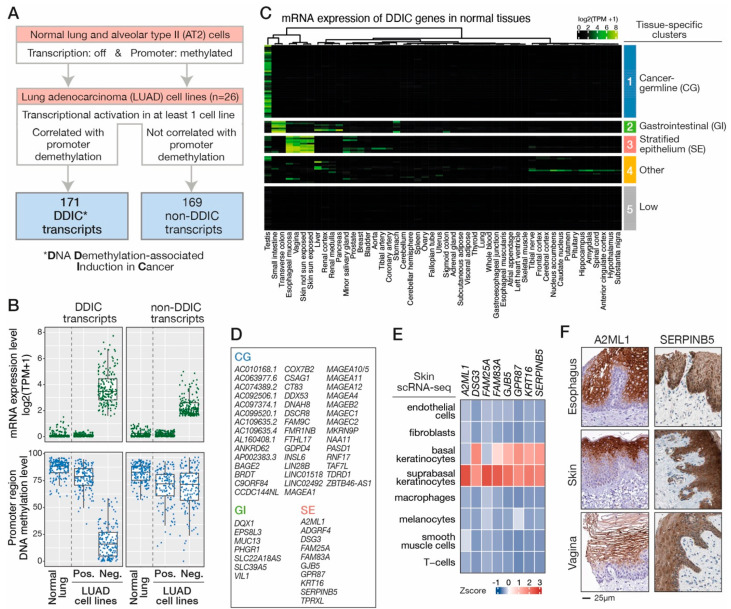
DNA demethylation in LUAD cell lines is associated with transcriptional induction of multiple tissue-specific gene expression programs. (**A**) Workflow for the identification of genes ex-hibiting DNA demethylation-associated induction (DDIC genes) in LUAD cell lines. (**B**) Levels of mRNA expression (RNA-seq) and promoter region methylation (Methyl-seq) of isolated DDIC and control non-DDIC transcripts were evaluated in normal lung and LUAD cell lines. For each tran-script, LUAD cell lines were divided in two subgroups according to the status of expression of the transcript (Positive or Negative). Dots represent mean mRNA level (green) and mean promoter methylation level (blue) of a single transcript in the corresponding subgroup of LUAD cell lines. (**C**) Median expression of DDIC genes in normal tissue samples (GTEx). Unsupervised hierarchical clus-tering was used to categorize DDIC genes according to their specificity of expression in normal tissue samples. (**D**) List of DDIC genes belonging to CG, GI and SE categories. (**E**) single-cell RNA-seq data (HPA) were used to determine relative expression levels of SE-DDIC genes in cells com-posing the skin tissue. (**F**) Immunohistochemical images from the HPA, which were available for two SE-DDIC proteins (A2ML1 and SERPINB5), show expression of these proteins in the stratified epithelium of the esophagus, skin and vagina.

In order to identify transcripts that are induced in lung cancer cells in association with promoter DNA demethylation, we conducted an integrative analysis of transcriptomic and methylomic datasets from LUAD cell lines (*n* = 26, DBTSS, Table S1), normal lung (*n* = 1, Roadmap Epigenomics) and lung AT2 cells (*n* = 1, [26]). A selection procedure was applied, using the following criteria (Figure 1A): (i) transcripts are not expressed and their promoter region is methylated in normal lung and AT2 cells; (ii) their expression is observed in a fraction of the LUAD cell lines (at least one cell line) and is inversely correlated with their promoter DNA methylation level; (iii) accuracy of expression specificity, TSS position, and the structure of the transcripts was validated after visualization of RNA-seq data with the Integrative Genome Viewer (IGV). This led to a final list of 171 transcripts that show transcriptional repression and promoter DNA methylation in normal lung and AT2 cells, but are transcriptionally induced in association with promoter demethylation in LUAD cell lines (Figure 1A,B; Table S2). Transcripts with this profile were qualified as “DDIC“ (*DNA Demethylation-associated Induction in Cancer*). Of note, transcripts that showed ectopic activation in LUAD cell lines, but without coincident loss of promoter DNA methylation (*n* = 169) were retained as a control group for subsequent analyses, and termed non-DDIC (*non-DNA Demethylation-associated Induction in Cancer*, Figure 1A,B; Table S2).

Among the 171 DDIC transcripts, 131 (77%) corresponded to previously referenced transcript variants of known genes, 19 (11%) to unreferenced transcript variants of known genes and 22 (12%) to unreferenced transcripts originating from previously undescribed genes (Figure S1). Together, these analyses led to the identification of a set of transcripts that are induced in LUAD cells in association with DNA demethylation of their promoter region.

### 3.2. DDIC Genes Belonging to Tissue-Specific Expression Programs

We next determined the pattern of expression of DDIC transcripts among normal tissues. To this end, we explored RNA-seq data from a series of normal human tissues (GTEx). To avoid confounding interpretations which may occur from genes harboring multiple transcript variants, only DDIC genes producing a single transcript were retained for the analysis (*n* = 103). Unsupervised hierarchical clustering identified three clusters of DDIC genes exhibiting restricted expression among normal tissues. The largest cluster of DDIC genes (Cluster 1, *n* = 44) displayed specific or preferential expression in testis (Figure 1C,D), and more specifically in testicular germ cells according to single cell RNA-seq data (Figure S2). Many of these genes corresponded to previously characterized CG genes. Another cluster of DDIC genes (Cluster 2, *n* = 7) displayed maximal expression in the small intestine and colon (Figure 1C,D), suggesting that DNA demethylation might contribute to induce a gastrointestinal (GI) gene expression program in LUAD cells. A third cluster of DDIC genes was identified (Cluster 3, *n* = 10), which showed preferential expression in esophagus, skin and vagina (Figure 1C,D). A common characteristic of these seemingly unrelated tissues is that they are composed of a multilayered stratified epithelium [44]. Consistently, single-cell RNA-seq (skin) and immunohistochemical data (esophagus, skin and vagina), which were available from the Human Protein Atlas, revealed specific expression of genes and proteins belonging to cluster 3 in the epithelial cell layers (Figure 1E,F). This suggested therefore that DDIC genes in Cluster 3 belong to an expression program associated with the formation of stratified epithelia (SE). Notably, expression of the lung alveolar cell marker *NKX2-1* was detected in tumors expressing GI- or SE-DDIC genes, indicating that these genes do not define subgroups of tumors originating from a different type of normal precursor cells (Figure S2). Finally, DDIC genes that did not belong to cluster 1, 2 or 3, showed either disparate patterns of expression among analyzed tissues (Other, *n* = 16), or expression levels that were below the defined threshold (<2 TPM) in all analyzed tissues (Low, *n* = 26).

Together, these observations suggested that DNA demethylation in tumor cells activates not only a germline expression program (CG), but might also be associated with induction of genes belonging to a gastrointestinal (GI) and a stratified epithelium (SE) transcription program.

### 3.3. DDIC Promoters Show Tissue- and Cell Type-Specific DNA Demethylation

**Figure 2:**
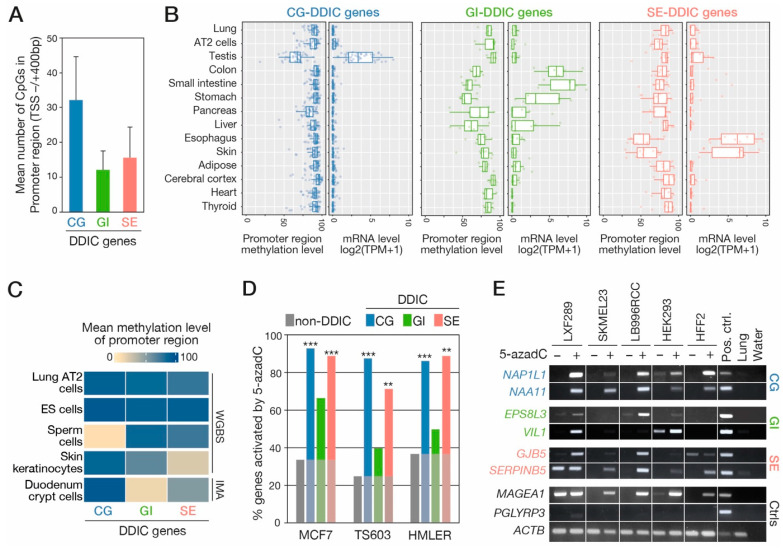
Promoter DNA methylation is involved in the regulation of DDIC genes of the CG, GI and SE clusters. (**A**) Mean number of CpGs in the promoter region of DDIC genes of the indicated cate-gories (mean ± *SD*). (**B**) Transcriptomic and methylomic data from the ENCODE database were used to compare mean levels of mRNA expression and promoter methylation of DDIC genes in a panel of normal tissues. Each dot represents the value for a single DDIC gene of the indicated category. (**C**) Publicly available methylomic data (whole genome bisulfite sequencing, WGBS; Infinium Me-thylation Assay, IMA) were examined to compare mean promoter methylation levels of CG-, GI- and SE-DDIC gene clusters in the indicated cell types. (**D**) RNA-seq data obtained from tumor cell lines (MCF7, TS603) and a mammary epithelial cell culture (HMLER) exposed to 5-azadC were ex-plored, and percentages of genes in each DDIC category that were significantly upregulated by the demethylating agent were measured, and compared to non-DDIC genes. Fischer’s exact test. ***: *p* < 0.001; **: *p* < 0.01. (**E**) RT-PCR experiments were performed to evaluate induction of indicated DDIC genes in 5-azadC-treated tumor cell lines or normal fibroblast culture (HFF2). Control genes included a previously described CG gene (*MAGEA1*), a non-DDIC gene (*PGLYRP3*), and a ubiqui-tously expressed gene (*ACTB*).

Genes regulated by DNA methylation are expected to exhibit matching patterns of expression and promoter demethylation in normal tissues. We searched to determine if this is the case for DDIC genes of the CG, GI, and SE clusters. First, the mean number of CpG sites comprised in the promoter region (TSS −/+ 400 bp) of DDIC genes was calculated (Figure 2A). DDIC genes in the GI and SE categories showed a lower density of CpGs in their promoter region, as compared with CG-DDIC genes, but nevertheless contained a mean amount of 14 CpGs within the 800 bp segment. Analysis of transcriptomic and methylomic data of normal human tissues (ENCODE database) revealed that DDIC genes categorized in the CG, GI, or SE clusters exhibited reduced DNA methylation levels of their promoter region in the tissues where they are expressed, thereby supporting a link between DNA demethylation and expression for these genes (Figure 2B).

We further explored the relationship between DNA demethylation and activation of CG, GI and SE transcription programs at the cellular level, by analyzing methylomic data derived from specific cell types, including human lung AT2 cells, embryonic stem cells, spermatozoa, skin keratinocytes and duodenum crypt cells (Figure 2C). In AT2 and embryonic stem (ES) cells, the promoter region of all three categories of DDIC genes exhibited comparably high levels of DNA methylation. In spermatozoa, as expected, the mean promoter DNA methylation levels of CG genes appeared much lower than that of the two other gene categories. In skin keratinocytes, which compose the stratified epithelium of the epidermis, the lowest level of DNA methylation was instead observed for DDIC genes belonging to the SE cluster. Genes of the GI cluster, on the other hand, showed lowest DNA methylation levels in intestinal (duodenum) crypt cells. Together these results revealed that DDIC genes of the CG, GI and SE categories exhibit highly distinct DNA demethylation patterns among normal cell types, and that these match with their tissue-specific expression profiles.

### 3.4. Experimental Evaluation of the Role of DNA Methylation in Regulating DDIC Transcripts

In order to establish a direct role of DNA demethylation in the transcriptional induction of DDIC transcripts, we decided to explore RNA-seq data obtained from human cell lines that had been exposed to the DNA demethylating agent 5-aza-deoxycytidine (5-azadC). The analyzed datasets derived from three different cell lines: the TS603 glioblastoma cell line, the MCF7 breast cancer cell line, and the HMLER transformed mammary epithelial cell line [34,45,46]. We evaluated the frequency of transcriptional induction of DDIC transcripts of the CG, GI, and SE categories upon 5-azadC treatment in these cells (Figure 2D, Table S2). For comparison, we also examined the impact of 5-azadC on non-DDIC transcripts, the expression of which was not correlated with promoter demethylation in LUAD cells (Figure 1A,B). Contrasting with the low proportion (25–37%) of non-DDIC transcripts exhibiting upregulation following 5-azadC, a large proportion (86–93%) of DDIC transcripts in the CG category showed significant induction in treated cells (Figure 2D). This was consistent with the well demonstrated role of DNA methylation in the regulation of CG genes. Interestingly, DDIC transcripts of the SE group also showed a high frequency of induction upon 5-azadC treatment (71–89%), thereby supporting an important role of DNA methylation in their regulation. For DDIC transcripts of the GI category, we observed an intermediate frequency of 5-azadC induction (40–67%), suggesting a less direct impact of DNA methylation on their regulation.

To verify the validity of these in silico observations, we conducted 5-azadC induction experiments in four tumor cell lines, as well as in normal cultured fibroblasts, and assessed the expression of several DDIC genes by RT-PCR. The results confirmed that 5-azadC treatment was more often associated with induction of CG- and SE-DDIC genes, than with GI-DDIC genes (Figure 2D). Contrastingly, 5-azadC had very little effect on the expression of a control non-DDIC gene. Together, the results indicate that DNA methylation acts as an essential component in the transcriptional regulation of DDIC genes belonging to the CG and SE categories, whereas it appeared to play a more auxiliary role in the control of expression of GI-DDIC genes.

### 3.5. Validating DNA Demethylation-Associated Induction of DDIC Genes In Vivo in LUAD Tumors

**Figure 3:**
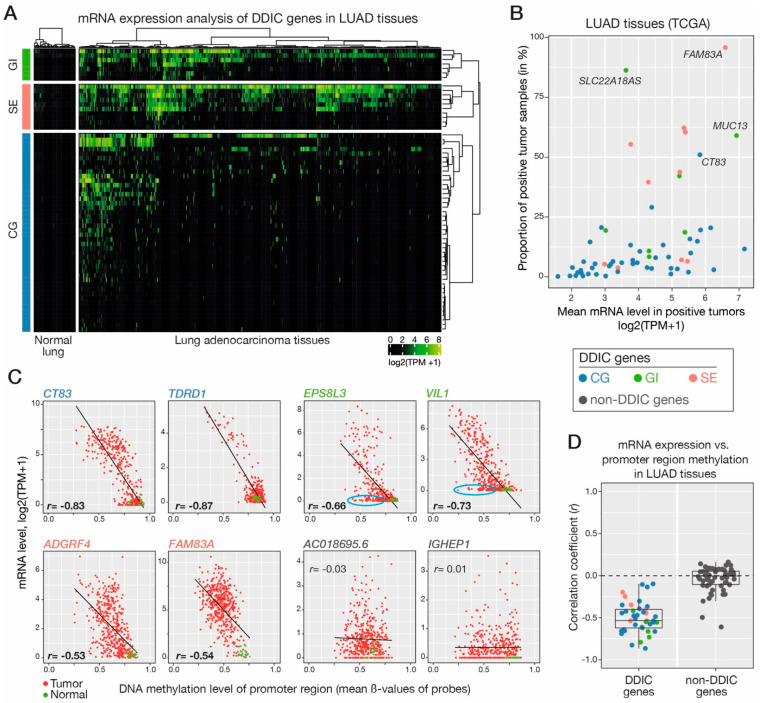
DDIC gene clusters are induced in vivo in LUAD tissues in association with promoter demethylation. (**A**) RNA-seq from TCGA were explored to evaluate expression levels of CG, GI and SE gene clusters in LUAD (*n* = 510) and normal lung tissues (*n* = 58). A heatmap was generated, where samples are clustered according to DDIC gene expression profiles. (**B**) Frequency of tran-scriptional induction in LUAD samples, and mRNA level in positive LUAD samples (each dot rep-resents a single gene with the indicated color code for categories). (**C**) Correlations between mRNA expression and promoter demethylation of DDIC genes in LUAD tissues: representative plots are shown, including two non-DDIC genes (red dots: tumor samples, green dots: normal lung tissues). Pearson’s correlation coefficients (*r*) and linear regression lines are shown. Tumor samples showing demethylation of the gene promoter without transcriptional induction are encircled in blue. (**D**) Correlation coefficients, as determined in panel C, were obtained for all CG-, GI- and SE-DDIC genes and compared with that of non-DDIC genes.

We next searched to verify if the DDIC genes we identified in LUAD cell lines also become induced in vivo in LUAD tissue samples, and whether this is also correlated with promoter demethylation. We first explored RNA-seq datasets of The Cancer Genome Atlas (TCGA), which were obtained from large series of LUAD (*n* = 510) and normal lung (*n* = 58) tissue samples [47]. Analysis of the data confirmed absent or very low mRNA expression of all DDIC genes in normal lung tissues, and significant induction in multiple tumor samples (Figure 3A). Immunohistochemical data which were available for several of the corresponding proteins (Human Protein Atlas) indicated that induction of DDIC genes in LUAD was associated with expression of the protein (Figure S4). DDIC genes of the GI and SE clusters showed a tendency to be induced in a higher proportion of LUAD samples, as compared with CG genes (Figure 3A,B; Table S2). However, in the tumors where they were induced, all three categories of DDIC genes displayed similar ranges of mRNA expression levels (Figure 3B). Unsupervised hierarchical clustering did not reveal groups of tumors expressing exclusively one or another category of DDIC genes.

To investigate the role of DNA demethylation in the induction of CG-, GI- and SE-DDIC genes in LUAD tissues, we analyzed methylomic data (Infinium methylation assay, IMA) from the TCGA. IMA probes interrogating the methylation status of the promoter region were available for 48 out of the 61 CG-, GI, or SE-DDIC genes. For each of these genes, the mRNA expression level and promoter region DNA methylation level were compared in the different LUAD samples, and a correlation coefficient (Pearson) was calculated (Figure 3C,D; Table S2). Consistent with a role for DNA demethylation in transcriptional induction, DDIC genes generally displayed a negative correlation coefficient (mean = −0.51; Figure 3D). Contrastingly, control non-DDIC genes exhibited a mean correlation coefficient close to 0.

Together, these results confirm that induction of DDIC genes also occurs in vivo in LUAD tissues, and is associated with DNA demethylation of the promoter region. For several genes of the GI cluster, however, we observed that in a fraction of the tumors, mRNA expression remained absent or low despite DNA demethylation of the promoter region (Figure 3C), thereby suggesting that other factors might be necessary for transcriptional induction of these genes.

### 3.6. Global Genome Hypomethylation Is Associated with Local Demethylation of DDIC Genes

**Figure 4:**
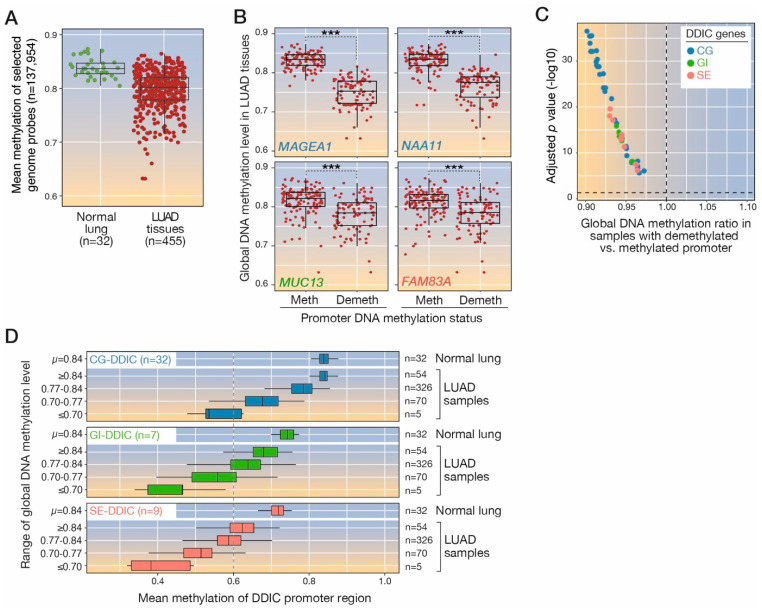
Focal DDIC promoter demethylation is associated with genome-wide loss of DNA meth-ylation in LUAD tissue samples. (**A**) Global DNA methylation estimates of LUAD and normal matching lung tissue samples of TCGA, based on the analysis of a selected pool of CpGs dispersed over the entire genome. (**B**) Tumor samples exhibiting either methylation (Meth) or demethylation (Demeth) of the indicated gene promoter were separated, and their level of global DNA methylation was compared (Student’s *t*-test, ***: *p* < 0.001). The results for four genes are illustrated. (**C**) Statistical values determined as shown in panel C, were obtained for all CG-, GI-, and SE-DDIC genes and plotted accordingly. (**D**) LUAD tissue samples were divided into subgroups according to their range of global genome methylation level. In each subgroup of samples, the mean level of methylation of gene promoters belonging to the indicated DDIC category was calculated, and is represented by a box plot. The same analysis was conducted in normal lung tissue samples.

It has been demonstrated previously for CG genes that local DNA demethylation of their promoter region in tumors coincides with a process of global genome demethylation [48,49,50]. Here we searched to determine if demethylation of DDIC gene promoters similarly correlates with genome-wide DNA hypomethylation in LUAD tumors. To this end, we established a procedure based on methylomic data from the TCGA to evaluate global genome methylation levels in LUAD tissue samples. We first searched to identify CpGs that are initially methylated in normal lung tissues. CpGs located on the X chromosome were ignored in order to avoid gender-related differences resulting from the X inactivation process in female cells. Among the >450 k CpG sites that are interrogated in TCGA datasets, we identified 137,954 CpGs that displayed high methylation levels (mean methylation ß value = 0.84) in all normal lung tissue samples (*n* = 32). We then calculated the mean level of methylation of all 137,954 CpGs in each LUAD sample, and used it as an estimator of overall genome methylation level. The results revealed that a majority (82%) of LUAD tissue samples exhibited discernable genome hypomethylation as compared with normal lung tissues (ß value < quantile 25 of normal tissues: 0.827; Figure 4A).

We next searched to determine if local DNA demethylation of DDIC gene promoters correlates with global genome hypomethylation. For each DDIC gene, LUAD samples were separated in two subgroups depending on the methylation status of the promoter region (Methylated or Unmethylated), and global genome methylation levels were compared between the two subgroups (Figure 4B). The results revealed a significant association between global genome hypomethylation and local promoter demethylation not only for CG genes, as expected, but also for all DDIC genes of the GI and SE categories (Figure 4C; Table S2). Demethylation of GI and SE gene promoters, however, was associated with a decrease in global DNA methylation that was generally less pronounced than for CG genes (Figure 4C). To further address this issue, we divided LUAD samples into four groups depending on the range of global DNA hypomethylation, and determined in every group the mean level of methylation of each category of DDIC gene promoters (Figure 4D). Consistent with previous data, the results showed that CG-DDIC gene promoters only become substantially demethylated (mean promoter ß-value < 0.6) in the LUAD samples that show a marked decrease in global DNA methylation (mean global ß value < 0.70). In comparison, GI- and SE-DDIC gene promoters already exhibited a similar level of local demethylation in tumors that only show an intermediate level of genome hypomethylation (mean global ß value < 0.77; Figure 4D). Moreover, we noted that in normal lung tissues, the mean methylation level of GI- and SE-DDIC gene promoters (0.74 and 0.72 mean ß values, respectively) was generally lower than that of CG-DDIC gene promoters (0.84; Figure 4D).

Together, these results indicate that local DNA demethylation of the promoters of all three categories of DDIC genes coincides with a process of global genome demethylation. However, compared with CG gene promoters, promoters of the GI and SE gene categories appear to be less extensively methylated in the normal lung tissue, and to already exhibit local DNA demethylation in tumors displaying a lower extent of genome hypomethylation.

### 3.7. Assessing the Role of Transcription Factors in the Activation of GI-DDIC Genes

**Figure 5:**
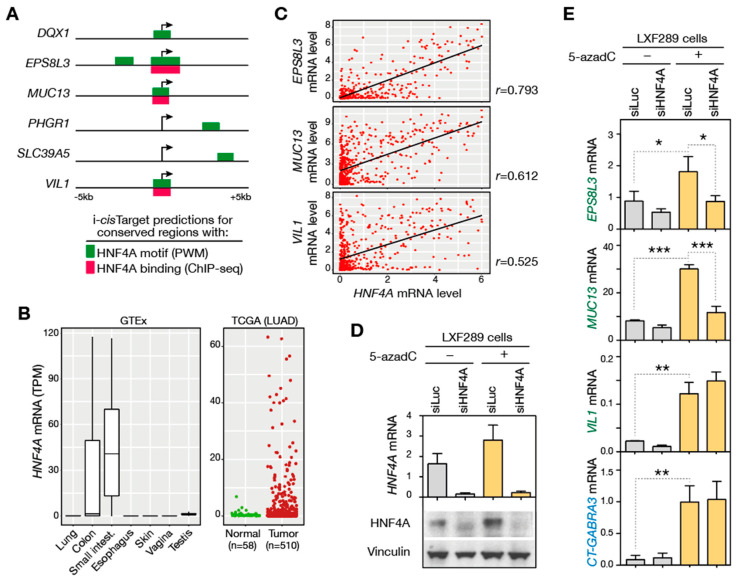
Transcription factor HNF4A contributes to transcriptional induction of DDIC genes of the GI cluster. (**A**) Schematic representation of i-*cis*Target predictions for GI-DDIC genes: conserved regions with HNF4A consensus binding motifs (based on position weight matrices, PWM) and ChIP-seq binding peaks (obtained from LoVo colon tumor cells) are depicted, with the broken arrow corresponding to the TSS. (**B**) RNA-seq data were analyzed to determine *HNF4A* expression in a panel of normal tissues (GTEx), as well as in LUAD and normal matching lung tissue samples (TCGA). (**C**) Expression (RNA-seq, log2(TPM+1)) of *HNF4a* was compared to that of *EPS8L3*, *MUC13*, and *VIL1* genes in LUAD samples (Pearson’s correlation coefficients (*r*) are indicated). (**D**) LXF289 LUAD cells were transfected with siRNAs against *HNF4a* (siHNF4A) or control siRNAs (siLuc), and then exposed to 5-azadC (+) or vehicle (−). HNF4A expression in the different cell groups was evaluated at the mRNA level by RT-qPCR (*n* = 3, mean ± *SEM*), and at the protein level by Western blot (Vinculin was used as loading control). (**E**) Cells exposed to these experimental conditions were also submitted to RT-qPCR analyses to evaluate levels of expression *EPS8L3*, *MUC13*, and *VIL1* genes (relative to *ACTB; CT-GABRA3* was used as a HNF4A-independent control gene). Values represent mean of 3 independent experiments ±*SEM* (One-way ANOVA, Tukey’s multiple comparison; ***: *p* <0.001; **: *p* <0.01; *: *p* < 0.05).

As mentioned before, the correlation between promoter demethylation and transcriptional activation that we observed for DDIC genes was less strict in the case of genes belonging to the GI cluster. In particular, we observed that in several LUAD samples, the expression of GI-DDIC genes remained absent despite demethylation of their promoter region (Figure 3C). This suggested that transcriptional induction of these genes might be conditioned by the presence of specific transcription factors. We therefore searched to identify transcription factors involved in the regulation of GI-DDIC genes. To this end we used i-*cis*Target, a prediction tool integrating sequence conservation, transcription factor binding motifs, and ChIP-seq data [51]. Analysis with i-*cis*Target revealed highly significant enrichment of consensus binding sites for Hepatocyte Nuclear Factor 4 Alpha (HNF4A), which were detected in conserved regions of 6 out of the 7 GI-DDIC genes (Figure 5A). Moreover, ChIP-seq data confirmed binding of HNF4A in the promoter region of 3 of these genes (*EPS8L3*, *MUC13*, *VIL1*; Figure 5A). HNF4A is a highly tissue-specific transcriptional activator, which plays a role in development of the liver and the gastrointestinal tract.

Analysis of GTEx RNA-seq datasets confirmed that *HNF4A* is highly expressed in colon and small intestine, and instead absent in other normal tissues, including lung (Figure 5B). Importantly, analysis of RNA-seq data from TCGA revealed ectopic activation of *HNF4A* in 23% of LUAD tissue samples (≥2 TPM, Figure 5B). To evaluate the role of HNF4A in the induction of *EPS8L3*, *MUC13*, and *VIL1* genes in LUAD, we first analyzed RNA-seq data to find out if their expression is correlated with that of *HNF4A*. The results showed positive correlation between *HNF4A* and all three GI-DDIC genes in LUAD samples, with highest correlation scores for *EPS8L3* and *MUC13* (Figure 5C). To further assess the potential role of HNF4A in regulating these genes, we tested the effect of its depletion on the ability to induce expression of *EPS8L3*, *MUC13* and *VIL1* genes with 5-azadC. Thus, LXF289 LUAD cells, which express HNF4A, were transfected with either control siRNAs (siLuc) or siRNAs directed against HNF4A (siHNF4A, Figure 5D and Figure S5), and were thereafter exposed to 5-azadC. The results revealed that induction of *MUC13* and *EPS8L3* (but not of *VIL1*) by 5-azadC was significantly impaired when HNF4A was downregulated (Figure 5E). Of note, HNF4A downregulation had instead no impact on 5-azadC induction of a CG gene (*CT-GABRA3*, Figure 5E). Together, these results illustrate the case where induction of GI-DDIC genes requires the presence of specific transcription factors, and identify HNF4A as one such factor.

### 3.8. Associating Genome Hypomethylation and DDIC Gene Activation with Tumor Grade and Patient Survival

**Figure 6:**
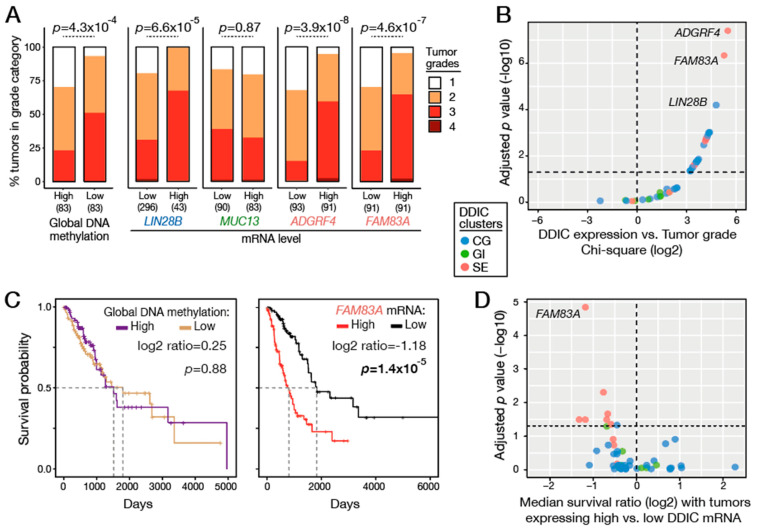
Associations of tumor grade and patient survival with genome hypomethylation and DDIC gene expression in LUAD. (**A**) Comparison of tumor grades in LUAD samples grouped ac-cording to global DNA methylation levels or mRNA levels of DDIC genes (low: q20; high: q80; number of samples are indicated below columns). Comparisons were performed using a Chi-squared test (adjusted *p*-value). (**B**) Tumor grade comparisons as described in A, were conducted for all CG-, GI- and SE-DDIC genes. Dots on the graph represent Chi-squared statistic vs. *p*-adjusted values. (**C**) Survival curves (Kaplan–Meier) of LUAD patients (TCGA) according to high or low global DNA methylation levels, and to high or low *FAM83A* mRNA levels (log-rank test). Dashed lines mark median survival time of the two subgroups, and the ratio between the two median times is indicated (log2). (**D**) Patient survival analyses, as described in C, were conducted for all CG-, GI- and SE-DDIC genes. Dots on the graph represent ratios of median survival in high/low subgroups (log2) and *p*-adjusted values.

It has been proposed that epigenetic alterations, including genome-wide DNA hypomethylation, might be associated with the increased plasticity of tumor cells, which often deviate from their original differentiation status. Tumor grading systems provide an estimate of the degree of cellular deviation, with the highest grade (4) corresponding to histological images showing cells with the most divergent appearance. Tumor grades for LUAD samples are available in TCGA datasets, and we first examined if global genome hypomethylation is indeed associated with loss of cellular differentiation, i.e., with a higher grade. The results revealed that global DNA hypomethylation in LUAD was significantly correlated with higher tumor grades (Figure 6A).

We then examined each DDIC gene of the CG, GI and SE clusters, to find out if their expression in LUAD also correlates with tumor grade. Interestingly, expression of DDIC genes of the CG and SE categories, but not of the GI category, was significantly associated with higher tumor grades (Figure 6A,B and Figure S2). Highest correlation scores were observed for *ADGRF4* and *FAM83A* genes of the SE category, and *LIN28B* of the CG category.

We also exploited clinical data from the TCGA to examine if genome hypomethylation and expression of DDIC genes are associated with reduced survival of LUAD patients. The results showed lack of correlation between genome hypomethylation and patient survival (Figure 6C). Instead, expression of several DDIC genes of the SE category, and most significantly of *FAM83A*, was significantly associated with reduced survival of LUAD patients (Figure 6D; Table S2).

## 4. Discussion

Most tumor cells show genome-wide losses of DNA methylation, and previous studies, including from our group, have shown that this epigenetic alteration is associated with the aberrant activation of a group of germline-specific (CG) genes [8]. Whether gene groups sharing other tissue specificities also become activated in association with genome demethylation in tumors remained an open question. Here, we investigated this issue by conducting an unbiased exploration of publicly available transcriptomic and methylomic datasets. Lung adenocarcinoma was chosen for this analysis, as datasets from both cell lines and tissues were available. Moreover, the normal precursor cells of this type of tumor has been identified (AT2 cells) [20], and transcriptomic and methylomic datasets from these cells were also available.

Our analyses confirm that CG genes constitute the dominant group of genes that become activated in tumor cells as a result of genome hypomethylation, since 43% of the genes that we identified as being induced in association with DNA demethylation (DDIC) showed specific expression in testicular germ cells. Importantly, however, we also identified two other tissue-specific gene clusters showing DNA demethylation-associated induction in lung tumor cell lines: one with specific expression in the gastrointestinal tract (GI), and the other displaying restricted expression in tissues comprising a stratified squamous epithelium (SE). Genes belonging to these two groups were however less abundant than CG genes, as they represented only 7% (GI) and 10% (SE) of all isolated DDIC genes.

Analysis of in vivo tumor samples confirmed aberrant induction of all three group of genes in LUAD tissues. CG genes, however, were activated in a lower proportion of tumor samples, as compared with GI- or SE-DDIC genes. Analysis of global DNA methylation levels indicates that CG genes only become activated in tumors that show extensive genome hypomethylation. In comparison, GI- and SE-DDIC genes already showed local promoter demethylation and transcriptional induction in tumors with less pronounced global genome hypomethylation. This is likely due to the fact that, compared with CG genes, GI- and SE-DDIC genes contain promoters displaying a lower amount of CpGs, and an initially lower level of methylation in normal tissues.

Transcriptional activation of DNA methylation-regulated genes relies not only on DNA demethylation, but also on the availability of transcription factors capable of inducing efficient promoter activity. It has been demonstrated for CG genes that such factors are present in most cells [52]. DNA demethylation is therefore a sufficient trigger for induction of these genes in most tumors. Our data suggest that this is also the case for SE-DDIC genes. A representative gene in this cluster is *SERPINB5*, the expression of which was previously shown to be regulated primarily by DNA methylation [53]. For GI-DDIC genes instead, we observed a more conditional link between DNA demethylation and transcriptional induction, which could be explained by a more restricted pattern of expression of activating transcription factors. Our data indicate that HNF4A constitutes one such factor, and show that aberrant induction of several GI-DDIC genes in LUAD tumors is dependent on ectopic upregulation of this liver- and gut-specific factor in addition to DNA demethylation. The precise mechanism by which HNF4A mediates induction of GI-DDIC genes in association with promoter DNA demethylation in LUAD tumors cells remains to be determined. It has been demonstrated previously that HNF4A is upregulated in a fraction of LUAD tumors, and contributes to a gene expression signature that characterizes a subtype of LUAD, which was defined as invasive mucinous adenocarcinoma [54]. It is likely that the genes we characterized here represent a restricted subgroup of this signature, for which transcriptional induction in LUAD relies not only on the presence of HNF4A, but also on promoter DNA demethylation.

Our results indicate that genome hypomethylation in LUAD is associated with loss of cellular differentiation, which is in line with previous observations in different tumor types [55,56]. Consistently, transcriptional induction of several DDIC genes was observed predominantly in poorly differentiated tumors. We found instead no association between the level of genome hypomethylation and the probability of survival of LUAD patients. Reduced patient survival was instead significantly associated with high expression of several genes belonging to the SE-DDIC cluster. Among these, *FAM83A* was most significantly associated with poor survival of LUAD patients. Ectopic expression of *FAM83A* has been previously described in different tumor types, including lung adenocarcinoma [57]. It has been demonstrated in a model of breast cancer that FAM83A acts downstream of the EGFR signaling and exerts oncogenic properties by promoting tumor growth, and conferring resistance to EGFR-tyrosine kinase inhibitors [58]. Mounting evidence suggests that *FAM83A* plays a predominant role in the development of a variety of tumors [59]. Our study now identifies promoter DNA demethylation as a critical contributor of aberrant upregulation of this important oncogene in cancer cells. Future experiments will be required to determine if other DDIC genes can act as cancer drivers in LUAD.

## 5. Conclusions

To conclude, our study reveals that genome hypomethylation in tumors is associated not only with aberrant activation of germline-specific genes, but also with ectopic induction of gene expression programs that are specific of defined somatic tissues. Several genes in these newly identified gene clusters were associated with poor survival of lung cancer patients. Moreover, our results reveal that these genes contain CpGs displaying highly contrasted methylation states in normal and tumoral lung tissues, and are therefore amenable to the development of DNA methylation biomarkers of prognostic significance. Several DNA methylation biomarkers have been successfully translated into clinical practice, but so far, these markers correspond to DNA sequences that show hypermethylation in tumor cells [60]. Our results are an incentive to explore the value of hypomethylated DNA sequences as cancer biomarkers.

## Data Availability

Accessions to archived datasets analyzed in this study are listed in Tables S1, S3 and S4.

## Supplementary Materials

The following supporting information can be downloaded at: https://www.mdpi.com/article/10.3390/cancers14041007/s1, Figure S1: Identified DDIC transcripts derive from referenced and unreferenced genes and transcript variants, Figure S2: CG-DDIC genes are predominantly expressed in testicular germ cells, Figure S3: GI- and SE-DDIC genes are co-expressed with NKX2-1, an AT2-specific marker, Figure S4: GI- and SE-DDIC proteins are ectopically expressed in LUAD tissue samples, Figure S5: Raw images of western-blot films corresponding to Figure 5D, Table S1: Methylomic and transcriptomic datasets of LUAD cell lines used in this study, Table S2: CG-, GI- and SE-DDIC gene list, Table S3: Methylomic and transcriptomic datasets of normal cells and tissues, Table S4: Transcriptomic datasets of cell lines treated with 5-azadC, Table S5: Antibodies and tissue section codes from HPA, Table S6: siRNAs used for transfection experiments, Table S7: Primer sequences, PCR and qPCR reagents and conditions.
