# Supplementary material for: Identification of Tissue-Specific Gene Clusters Induced by DNA Demethylation in Lung Adenocarcinoma: More Than Germline Genes
Source: Cancers (Basel). 2022 Feb 16;14(4):1007. doi: 10.3390/cancers14041007 (PMC8870412; doi:10.3390/cancers14041007)
Supplement: Supplementary file 1 [file cancers-14-01007-s001.zip › cancers-1546266-supplementary.pdf]

cancers MDPI

Supplementary materials, Diacofotaki et al.

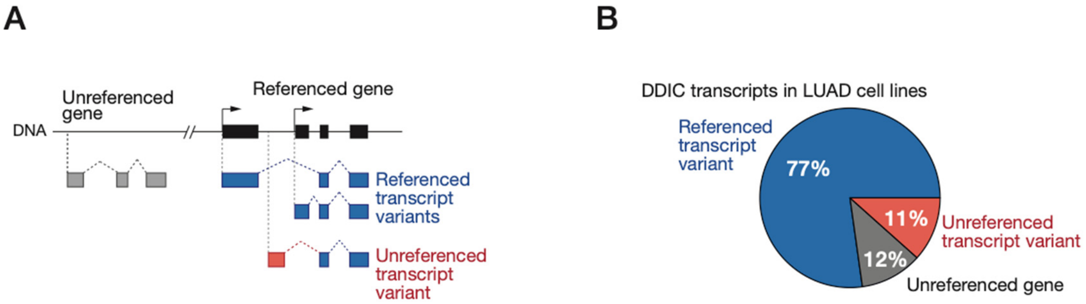

Figure S1. Identified DDIC transcripts derive from referenced and unreferenced genes and— transcript variants. A) DDIC transcripts which were identified in our screening of RNA-seq data were compared to the annotated reference genome. The meaning of referenced vs unreferenced genes and transcript variants is shown below. B) Proportions of DDIC in each of these categories is presented.

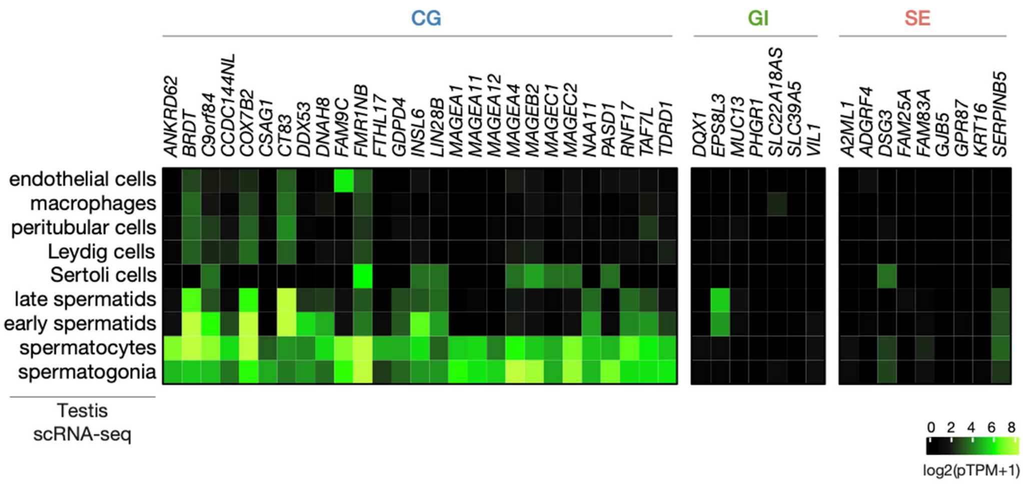

Figure S2. CG-DDIC genes are predominantly expressed in testicular germ cells. Heatmap representing the expression level of CG-, GI- and SE-DDIC genes in cells constituting the tissue of the testis (scRNA-seq data from the HPA; pTPM: protein Transcripts Per Million).

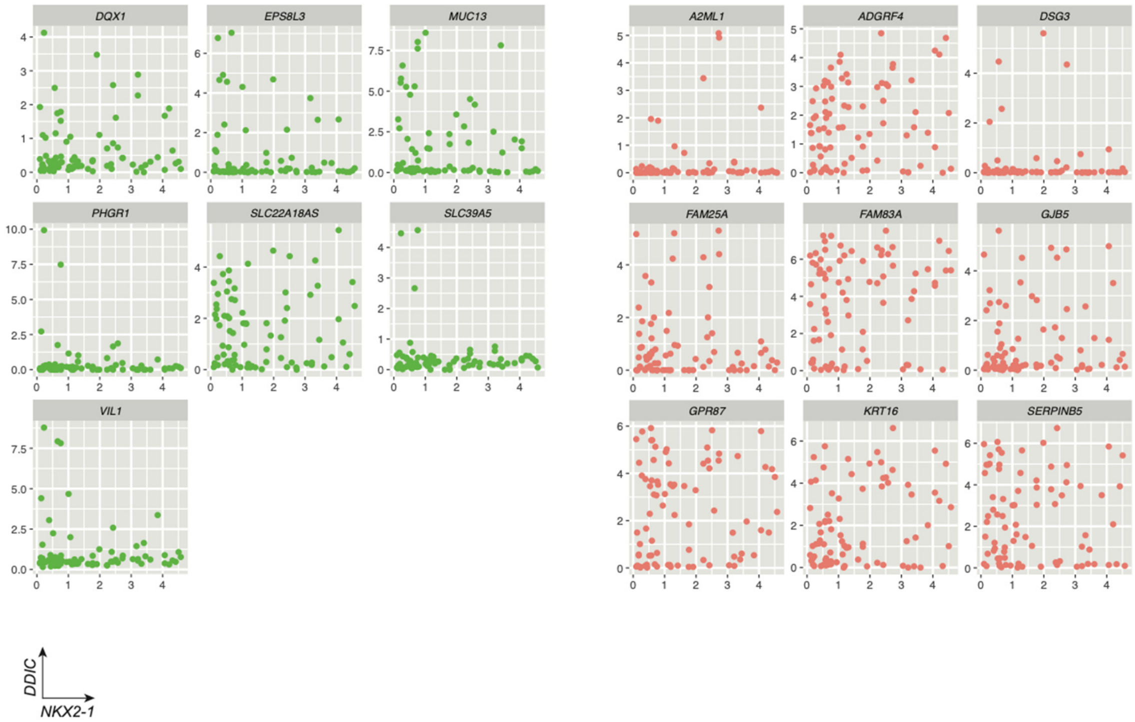

Figure S3. GI- and SE-DDIC genes are co-expressed with NKX2-1, an AT2-specific marker. Each dot represents a LUAD cell line for which DDIC gene and NKX2-1 expressions are reported on the y and x axis respectively. The DDIC gene name is reported on top of each graph. Expression data (in log2(TPM+1)) were obtained via DepMap portal (https://depmap.org/portal/) and correspond to 19Q3 release.

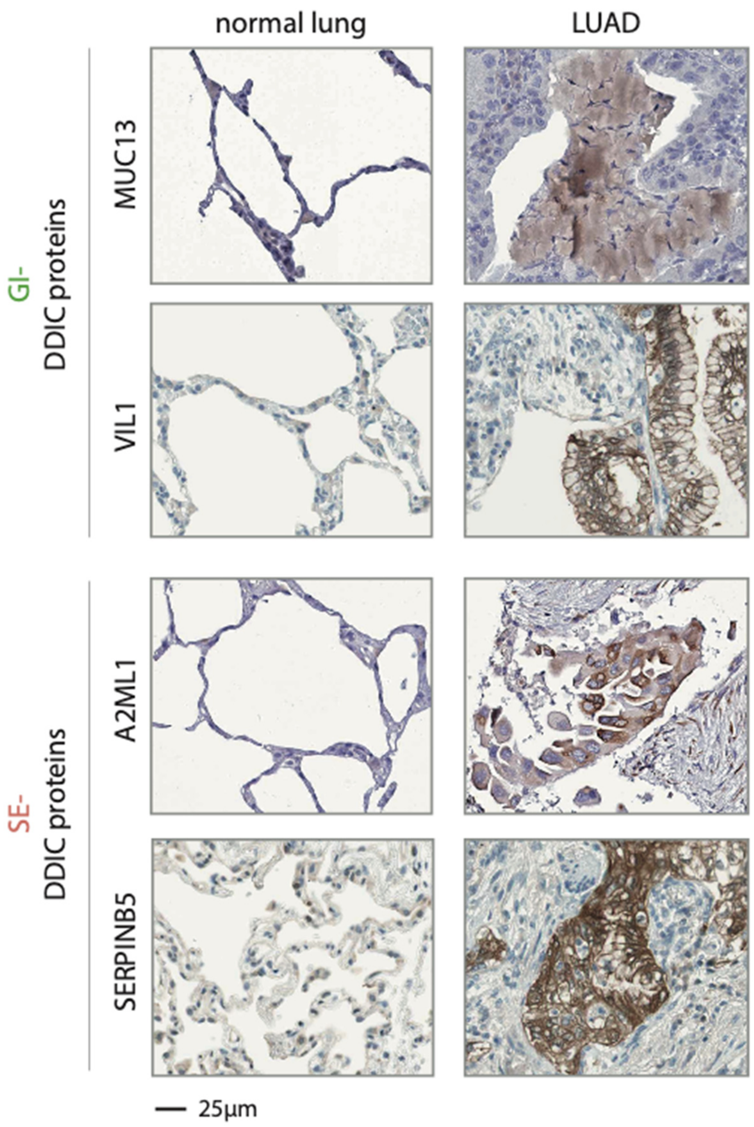

Figure S4. GI- and SE-DDIC proteins are ectopically expressed in LUAD tissue samples. Immunohistochemical images from the HPA of normal lung and LUAD tissue samples stained with antibodies targeting the above-mentioned GI- and SE-DDIC proteins. Antibodies and tissue codes are listed in supplementary Table S5 and were selected using the same criteria as described in the material and methods section.

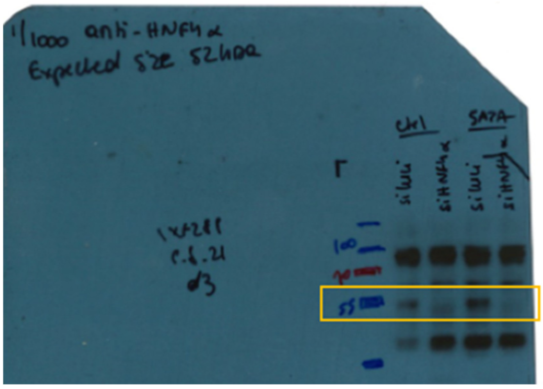

A

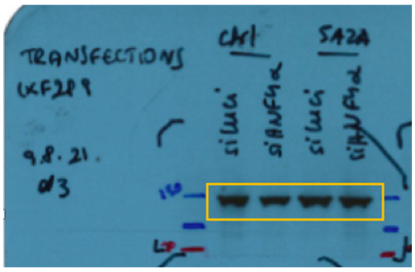

B

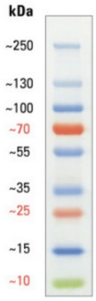

C

Condition	Treatment	Lanes	VCL area	HNF4A area	HNF4A/VCL area ratio	% siLuc condition
siLuc	ctrl	1	42345,936	16245,108	0,384	100
siHNF4a	ctrl	2	35563,016	11172,915	0,314	81,9
siLuc	5-azadC	3	42110,714	35563,057	0,845	100
siHNF4a	5-azadC	4	38624,844	5159,388	0,134	15,8

D

Figure S5. Raw images of western-blot films corresponding to Fig. 5D. Original uncropped images of HNF4A (A) and VCL (B) detections. Yellow rectangles delineate the image parts shown in the main figure. C) Protein size bands of the marker used (in kDa; PageRuler ThermoFischer). Protein sizes are indicated in red and blue on the film images. D) Signal quantifications of the lanes of interest (A) using ImageJ software (v1.43).

Table S1. Methylomic and transcriptomic datasets of LUAD cell lines used in this study.

Sample	Type	Pathology	Accession	File	Assay	BioProject	Method	Layout	Platform	Reference
A427	cell line	lung adenocarcinoma	DRR016652	FastQ	methylation	PRJDB1928	Bisulfite-sequencing	paired	Illumina HiSeq 2500	Suzuki et al. 2014
A549	cell line	lung adenocarcinoma	DRR016653	FastQ	methylation	PRJDB1928	Bisulfite-sequencing	paired	Illumina HiSeq 2500	Suzuki et al. 2014
ABC1	cell line	lung adenocarcinoma	DRR016654	FastQ	methylation	PRJDB1928	Bisulfite-sequencing	paired	Illumina HiSeq 2500	Suzuki et al. 2014
H1299	cell line	lung adenocarcinoma	DRR016655	FastQ	methylation	PRJDB1928	Bisulfite-sequencing	paired	Illumina HiSeq 2500	Suzuki et al. 2014
H1437	cell line	lung adenocarcinoma	DRR016656	FastQ	methylation	PRJDB1928	Bisulfite-sequencing	paired	Illumina HiSeq 2500	Suzuki et al. 2014
H1648	cell line	lung adenocarcinoma	DRR016657	FastQ	methylation	PRJDB1928	Bisulfite-sequencing	paired	Illumina HiSeq 2500	Suzuki et al. 2014
H1650	cell line	lung adenocarcinoma	DRR016658	FastQ	methylation	PRJDB1928	Bisulfite-sequencing	paired	Illumina HiSeq 2500	Suzuki et al. 2014
H1703	cell line	lung adenocarcinoma	DRR016659	FastQ	methylation	PRJDB1928	Bisulfite-sequencing	paired	Illumina HiSeq 2500	Suzuki et al. 2014
H1819	cell line	lung adenocarcinoma	DRR016660	FastQ	methylation	PRJDB1928	Bisulfite-sequencing	paired	Illumina HiSeq 2500	Suzuki et al. 2014
H1975	cell line	lung adenocarcinoma	DRR016661	FastQ	methylation	PRJDB1928	Bisulfite-sequencing	paired	Illumina HiSeq 2500	Suzuki et al. 2014
H2126	cell line	lung adenocarcinoma	DRR016662	FastQ	methylation	PRJDB1928	Bisulfite-sequencing	paired	Illumina HiSeq 2500	Suzuki et al. 2014
H2228	cell line	lung adenocarcinoma	DRR016663	FastQ	methylation	PRJDB1928	Bisulfite-sequencing	paired	Illumina HiSeq 2500	Suzuki et al. 2014
H2347	cell line	lung adenocarcinoma	DRR016664	FastQ	methylation	PRJDB1928	Bisulfite-sequencing	paired	Illumina HiSeq 2500	Suzuki et al. 2014
H322	cell line	lung adenocarcinoma	DRR016665	FastQ	methylation	PRJDB1928	Bisulfite-sequencing	paired	Illumina HiSeq 2500	Suzuki et al. 2014
II18	cell line	lung adenocarcinoma	DRR016666	FastQ	methylation	PRJDB1928	Bisulfite-sequencing	paired	Illumina HiSeq 2500	Suzuki et al. 2014
LC2ad	cell line	lung adenocarcinoma	DRR016667	FastQ	methylation	PRJDB1928	Bisulfite-sequencing	paired	Illumina HiSeq 2500	Suzuki et al. 2014
PC14	cell line	lung adenocarcinoma	DRR016668	FastQ	methylation	PRJDB1928	Bisulfite-sequencing	paired	Illumina HiSeq 2500	Suzuki et al. 2014
PC3	cell line	lung adenocarcinoma	DRR016669	FastQ	methylation	PRJDB1928	Bisulfite-sequencing	paired	Illumina HiSeq 2500	Suzuki et al. 2014
PC7	cell line	lung adenocarcinoma	DRR016670	FastQ	methylation	PRJDB1928	Bisulfite-sequencing	paired	Illumina HiSeq 2500	Suzuki et al. 2014
PC9	cell line	lung adenocarcinoma	DRR016671	FastQ	methylation	PRJDB1928	Bisulfite-sequencing	paired	Illumina HiSeq 2500	Suzuki et al. 2014
RERF_LC_Ad1	cell line	lung adenocarcinoma	DRR016672	FastQ	methylation	PRJDB1928	Bisulfite-sequencing	paired	Illumina HiSeq 2500	Suzuki et al. 2014
RERF_LC_Ad2	cell line	lung adenocarcinoma	DRR016673	FastQ	methylation	PRJDB1928	Bisulfite-sequencing	paired	Illumina HiSeq 2500	Suzuki et al. 2014
RERF_LC_KJ	cell line	lung adenocarcinoma	DRR016674	FastQ	methylation	PRJDB1928	Bisulfite-sequencing	paired	Illumina HiSeq 2500	Suzuki et al. 2014
RERF_LC_MS	cell line	lung adenocarcinoma	DRR016675	FastQ	methylation	PRJDB1928	Bisulfite-sequencing	paired	Illumina HiSeq 2500	Suzuki et al. 2014
RERF_LC_OK	cell line	lung adenocarcinoma	DRR016676	FastQ	methylation	PRJDB1928	Bisulfite-sequencing	paired	Illumina HiSeq 2500	Suzuki et al. 2014
VMRC_LCD	cell line	lung adenocarcinoma	DRR016677	FastQ	methylation	PRJDB1928	Bisulfite-sequencing	paired	Illumina HiSeq 2500	Suzuki et al. 2014
A427	cell line	lung adenocarcinoma	DRR016694	FastQ	expression	PRJDB2256	bulk RNA-seq	paired	Illumina HiSeq 2500	Suzuki et al. 2014
A549	cell line	lung adenocarcinoma	DRR016695	FastQ	expression	PRJDB2256	bulk RNA-seq	paired	Illumina HiSeq 2500	Suzuki et al. 2014
ABC1	cell line	lung adenocarcinoma	DRR016696	FastQ	expression	PRJDB2256	bulk RNA-seq	paired	Illumina HiSeq 2500	Suzuki et al. 2014

H1299	cell line	lung adenocarcinoma	DRR016697	FastQ	expression	PRJDB2256	bulk RNA-seq	paired	Illumina HiSeq 2500	Suzuki et al. 2014
H1437	cell line	lung adenocarcinoma	DRR016698	FastQ	expression	PRJDB2256	bulk RNA-seq	paired	Illumina HiSeq 2500	Suzuki et al. 2014
H1648	cell line	lung adenocarcinoma	DRR016699	FastQ	expression	PRJDB2256	bulk RNA-seq	paired	Illumina HiSeq 2500	Suzuki et al. 2014
H1650	cell line	lung adenocarcinoma	DRR016700	FastQ	expression	PRJDB2256	bulk RNA-seq	paired	Illumina HiSeq 2500	Suzuki et al. 2014
H1703	cell line	lung adenocarcinoma	DRR016701	FastQ	expression	PRJDB2256	bulk RNA-seq	paired	Illumina HiSeq 2500	Suzuki et al. 2014
H1819	cell line	lung adenocarcinoma	DRR016702	FastQ	expression	PRJDB2256	bulk RNA-seq	paired	Illumina HiSeq 2500	Suzuki et al. 2014
H1975	cell line	lung adenocarcinoma	DRR016703	FastQ	expression	PRJDB2256	bulk RNA-seq	paired	Illumina HiSeq 2500	Suzuki et al. 2014
H2126	cell line	lung adenocarcinoma	DRR016704	FastQ	expression	PRJDB2256	bulk RNA-seq	paired	Illumina HiSeq 2500	Suzuki et al. 2014
H2228	cell line	lung adenocarcinoma	DRR016705	FastQ	expression	PRJDB2256	bulk RNA-seq	paired	Illumina HiSeq 2500	Suzuki et al. 2014
H2347	cell line	lung adenocarcinoma	DRR016706	FastQ	expression	PRJDB2256	bulk RNA-seq	paired	Illumina HiSeq 2500	Suzuki et al. 2014
H322	cell line	lung adenocarcinoma	DRR016707	FastQ	expression	PRJDB2256	bulk RNA-seq	paired	Illumina HiSeq 2500	Suzuki et al. 2014
II18	cell line	lung adenocarcinoma	DRR016708	FastQ	expression	PRJDB2256	bulk RNA-seq	paired	Illumina HiSeq 2500	Suzuki et al. 2014
LC2ad	cell line	lung adenocarcinoma	DRR016709	FastQ	expression	PRJDB2256	bulk RNA-seq	paired	Illumina HiSeq 2500	Suzuki et al. 2014
PC14	cell line	lung adenocarcinoma	DRR016710	FastQ	expression	PRJDB2256	bulk RNA-seq	paired	Illumina HiSeq 2500	Suzuki et al. 2014
PC3	cell line	lung adenocarcinoma	DRR016711	FastQ	expression	PRJDB2256	bulk RNA-seq	paired	Illumina HiSeq 2500	Suzuki et al. 2014
PC7	cell line	lung adenocarcinoma	DRR016712	FastQ	expression	PRJDB2256	bulk RNA-seq	paired	Illumina HiSeq 2500	Suzuki et al. 2014
PC9	cell line	lung adenocarcinoma	DRR016713	FastQ	expression	PRJDB2256	bulk RNA-seq	paired	Illumina HiSeq 2500	Suzuki et al. 2014
RERF_LC_Ad1	cell line	lung adenocarcinoma	DRR016714	FastQ	expression	PRJDB2256	bulk RNA-seq	paired	Illumina HiSeq 2500	Suzuki et al. 2014
RERF_LC_Ad2	cell line	lung adenocarcinoma	DRR016715	FastQ	expression	PRJDB2256	bulk RNA-seq	paired	Illumina HiSeq 2500	Suzuki et al. 2014
RERF_LC_KJ	cell line	lung adenocarcinoma	DRR016716	FastQ	expression	PRJDB2256	bulk RNA-seq	paired	Illumina HiSeq 2500	Suzuki et al. 2014
RERF_LC_MS	cell line	lung adenocarcinoma	DRR016717	FastQ	expression	PRJDB2256	bulk RNA-seq	paired	Illumina HiSeq 2500	Suzuki et al. 2014
RERF_LC_OK	cell line	lung adenocarcinoma	DRR016718	FastQ	expression	PRJDB2256	bulk RNA-seq	paired	Illumina HiSeq 2500	Suzuki et al. 2014
VMRC_LCD	cell line	lung adenocarcinoma	DRR016719	FastQ	expression	PRJDB2256	bulk RNA-seq	paired	Illumina HiSeq 2500	Suzuki et al. 2014

Table S2. CG-, GI- and SE-DDIC gene list.

Gene name	Cluster	Strand	Chr	CpG number	ENSG identifier	TSS	MCF7	TS603	HMLER	Positive tumors (%)	Mean TPM in positive tumors	Correlation coefficient	Ratio	Adjusted p-value	Chi-squared statistic	adjusted p-value	Ratio	Adjusted p-value
AC010168.1	CG	+	12	18	ENSG00000214772	14665655	yes	yes	NA	14.51	4.87	NA	NA	NA	12.04	0.0192	0.82	0.8783
AC063977.6	CG	-	19	53	ENSG00000269072	51181966	yes	NA	NA	1.57	3.90	-0.35	0.92	7.00E-28	NA	NA	0.79	0.8783

AC074389.2	CG	+	7	38	ENSG000231476	1619444	yes	NA	no	3.92	18.10	-0.62	0.92	1.47E-29	3.38	0.4350	0.78	0.9338
AC092506.1	CG	+	1	24	ENSG000215869	104072983	yes	no	NA	3.92	3.07	-0.55	0.92	4.21E-24	1.72	0.7381	1.32	0.5351
AC097374.1	CG	-	2	24	ENSG000259848	94947341	yes	no	no	0.39	2.87	NA	NA	NA	NA	NA	NA	NA
AC099520.1	CG	-	5	23	ENSG000251574	105392970	no	no	NA	0.39	4.06	NA	NA	NA	NA	NA	NA	NA
AC109635.2	CG	+	11	59	ENSG000254518	50298579	yes	NA	NA	1.18	4.65	-0.40	0.97	2.33E-06	NA	NA	NA	NA
AC109635.4	CG	-	11	68	ENSG000255042	50298452	yes	no	no	3.33	34.70	-0.45	0.96	8.47E-09	2.32	0.6080	0.78	0.9455
AL160408.1	CG	+	1	9	ENSG000228044	234656125	yes	NA	yes	5.88	14.57	NA	NA	NA	3.50	0.2749	1.01	0.9184
ANKRD62	CG	+	18	55	ENSG000181626	12093843	yes	NA	yes	0.39	3.37	-0.47	0.95	3.89E-12	NA	NA	NA	NA
AP002383.3	CG	+	11	31	ENSG000256779	95038372	yes	NA	yes	2.16	9.42	-0.42	0.95	7.83E-14	NA	NA	1.71	0.8973
BAGE2	CG	+	21	30	ENSG000187172	10413532	yes	yes	no	5.49	5.51	NA	NA	NA	9.44	0.0452	2.04	0.7440
BRDT	CG	+	1	39	ENSG000137948	91949371	yes	no	yes	29.02	20.01	-0.66	0.96	2.59E-07	12.74	0.0160	1.59	0.1679
C9ORF84	CG	-	9	32	ENSG000165181	111795008	yes	yes	yes	20.59	6.43	NA	0.95	4.19E-10	13.28	0.0143	0.90	0.5449
CCDC144NL	CG	-	17	20	ENSG000205212	20868225	yes	NA	yes	1.76	3.53	NA	NA	NA	NA	NA	0.75	0.7236
COX7B2	CG	-	4	28	ENSG000170516	46909207	yes	yes	yes	13.33	25.62	-0.45	0.91	5.74E-33	18.33	0.0018	1.75	0.8783
CSAG1	CG	-	X	26	ENSG000198930	152733691	yes	yes	yes	20.39	70.18	-0.66	0.91	5.74E-33	20.22	0.0011	0.83	0.7236
CT83	CG	-	X	23	ENSG000204019	116462976	no	yes	NA	50.98	55.98	-0.83	0.96	8.93E-08	0.21	0.8996	0.90	0.7440
DDX53	CG	+	X	27	ENSG000184735	22999960	yes	yes	yes	3.73	5.14	-0.49	0.93	1.65E-22	10.18	0.0334	1.71	0.9711
DNAH8	CG	+	6	28	ENSG000124721	38723122	yes	yes	yes	0.20	2.01	-0.10	0.91	2.25E-36	NA	NA	NA	NA
DSCR8	CG	+	21	31	ENSG000198054	38121451	yes	yes	yes	8.04	21.78	NA	NA	NA	9.36	0.0452	0.80	0.8098
FAM9C	CG	-	X	34	ENSG000187268	13044602	yes	yes	yes	1.18	30.26	-0.30	0.97	8.33E-07	NA	NA	NA	NA

FMR1NB	CG	+	X	37	ENSG000176988	147981337	yes	yes	yes	1.96	43.87	-0.43	0.91	1.47E-29	NA	NA	0.47	0.7236
FTHL17	CG	-	X	59	ENSG000132446	31072041	yes	yes	yes	2.94	74.92	-0.69	0.94	3.94E-17	NA	NA	1.27	0.9455
GDPD4	CG	-	11	30	ENSG000178795	77301649	yes	yes	yes	0.39	7.11	-0.13	0.96	1.22E-08	NA	NA	NA	NA
INSL6	CG	-	9	50	ENSG000120210	5185639	yes	NA	yes	3.53	19.98	-0.55	0.94	7.48E-18	4.90	0.2750	0.70	0.9455
LIN28B	CG	+	6	18	ENSG000187772	104936616	yes	yes	yes	6.47	8.42	NA	NA	NA	27.77	6.61E-05	0.73	0.3389
LINC01518	CG	-	10	26	ENSG000233515	42691721	yes	no	yes	13.33	12.57	NA	NA	NA	16.43	0.0035	0.78	0.7236
LINC02492	CG	-	4	22	ENSG000250590	187672755	no	NA	NA	6.27	4.14	NA	NA	NA	9.27	0.0454	0.74	0.2798
MAGEA1	CG	+	X	23	ENSG000198681	153179284	yes	yes	yes	14.90	53.06	-0.58	0.90	2.23E-37	12.77	0.0160	0.73	0.0470
MAGEA10-MAGEA5	CG	-	X	35	ENSG000266560	152138493	NA	NA	NA	2.75	3.85	-0.48	0.91	2.66E-36	5.32	0.2462	0.64	0.1838
MAGEA11	CG	+	X	32	ENSG000185247	149712060	yes	yes	yes	5.49	13.24	NA	NA	NA	11.57	0.0220	0.70	0.3389
MAGEA12	CG	+	X	28	ENSG000213401	152733757	yes	yes	yes	19.61	56.72	-0.66	0.91	5.74E-33	20.69	0.0010	0.84	0.9455
MAGEA4	CG	+	X	23	ENSG000147381	151912495	yes	yes	yes	11.57	142.20	-0.44	0.91	9.70E-35	12.17	0.0192	0.69	0.7519
MAGEB2	CG	+	X	34	ENSG000099399	30215563	yes	yes	yes	10.00	46.08	-0.57	0.92	7.04E-25	19.38	0.0014	0.84	0.8783
MAGEC1	CG	+	X	27	ENSG000155495	141903894	yes	yes	yes	8.24	28.82	-0.51	0.91	3.09E-31	11.98	0.0192	0.84	0.9455
MAGEC2	CG	-	X	22	ENSG000046774	142205290	yes	yes	yes	15.88	45.32	-0.54	0.91	2.65E-36	20.99	0.0010	0.75	0.7236
MKRN9P	CG	-	12	23	ENSG000258128	87784568	yes	yes	no	4.51	7.74	-0.64	0.95	1.18E-11	5.31	0.2462	2.06	0.8783
NAA11	CG	-	4	27	ENSG000156269	79326061	yes	yes	yes	5.88	10.31	-0.53	0.92	5.02E-28	1.26	0.8049	4.86	0.8288
PASD1	CG	+	X	25	ENSG000166049	151563729	yes	yes	yes	3.53	30.31	-0.37	0.91	7.87E-30	0.64	0.8996	0.53	0.2723
RNF17	CG	+	13	37	ENSG000132972	24764169	yes	yes	yes	0.98	5.69	-0.39	0.91	1.29E-30	NA	NA	NA	NA

TAF7L	CG	-	X	29	ENSG00000102387	101293057	yes	yes	yes	6.86	13.18	-0.10	0.91	4.18E-33	11.10	0.0251	1.18	0.7236
TDRD1	CG	+	10	36	ENSG00000095627	114179270	yes	yes	yes	10.59	15.44	-0.86	0.96	6.63E-09	10.47	0.0306	1.86	0.1243
ZBTB46-AS1	CG	+	20	52	ENSG00000231208	63808076	yes	NA	yes	5.29	8.02	-0.73	0.92	6.87E-25	4.71	0.2796	0.84	0.8973
DQX1	GI	-	2	10	ENSG00000144045	74526191	yes	no	yes	19.41	7.11	-0.79	0.94	2.41E-13	1.60	0.7502	0.76	0.8783
EPS8L3	GI	-	1	12	ENSG00000198758	109763923	yes	NA	NA	18.63	40.76	-0.66	0.94	7.55E-14	2.62	0.5562	0.62	0.0512
MUC13	GI	-	3	10	ENSG00000173702	124934747	no	yes	yes	59.02	120.55	-0.61	0.96	7.91E-09	0.92	0.8734	0.80	0.2798
PHGR1	GI	+	15	7	ENSG00000233041	40351033	NA	NA	no	8.43	18.94	-0.42	0.94	1.56E-16	2.69	0.5544	0.78	0.7519
SLC22A18AS	GI	-	11	23	ENSG00000254827	2903490	no	yes	no	86.27	11.34	-0.54	0.96	7.16E-09	2.62	0.3693	1.38	0.7236
SLC39A5	GI	+	12	7	ENSG00000139540	56230063	no	no	no	10.78	18.86	-0.56	0.94	2.71E-14	0.61	0.8049	1.09	0.8783
VIL1	GI	+	2	11	ENSG00000127831	218419123	yes	no	yes	42.16	36.29	-0.73	0.94	3.27E-15	3.79	0.3775	1.19	0.8783
A2ML1	SE	+	12	10	ENSG00000166535	8822621	yes	no	NA	6.47	43.07	-0.45	0.94	9.07E-14	3.91	0.3693	0.70	0.1838
ADGRF4	SE	+	6	8	ENSG00000153294	47698580	yes	no	no	55.29	12.62	-0.53	0.95	1.17E-14	45.29	3.94E-08	0.62	0.0322
DSG3	SE	+	18	12	ENSG00000134757	31447741	NA	yes	yes	7.06	37.95	-0.33	0.95	4.74E-12	11.03	0.0251	0.44	0.0322
FAM25A	SE	+	10	12	ENSG00000188100	87020294	no	NA	yes	3.73	9.43	-0.20	0.93	2.82E-20	18.10	0.0019	0.69	0.1243
FAM83A	SE	+	8	32	ENSG00000147689	123183158	yes	yes	yes	95.88	95.37	-0.53	0.96	6.47E-07	38.85	4.58E-07	0.44	0.0000
GJB5	SE	+	1	13	ENSG00000189280	34755047	yes	NA	yes	39.61	18.62	-0.35	0.93	9.98E-19	4.75	0.2796	0.66	0.0446
GPR87	SE	-	3	8	ENSG00000138271	151316820	yes	NA	yes	62.16	39.92	-0.40	0.96	3.43E-08	18.81	0.0016	0.40	0.0322
KRT16	SE	-	17	27	ENSG00000186832	41612899	yes	yes	yes	60.39	41.10	-0.25	0.94	1.13E-13	10.99	0.0251	0.59	0.0050
SERPINB5	SE	+	18	24	ENSG00000206075	63476958	yes	yes	yes	43.73	36.59	-0.25	0.93	7.97E-18	17.53	0.0023	0.63	0.0217
TPRXL	SE	+	3	11	ENSG00000180438	14017401	yes	yes	no	5.29	6.93	NA	NA	NA	0.81	0.8823	0.73	0.6652

Table S3. Methylomic and transcriptomic datasets of normal cells and tissues.

Sample	Type	Pathology	Accession	File	Assay	Genome built	Method	Layout	Platform	Reference
alveolar type II	primary cells	normal	SRR1773103	FastQ	methylation	not applicable	Whole genome bisulfite-seq	paired	Illumina HiSeq 2000	Zuber et al. 2016
sperm	primary cells	normal	GSM1127119	wig	methylation	hg19	Whole genome bisulfite-seq	paired	Illumina HiSeq 2000	Roadmap Epigenomics 2015
HUES64	primary cells	normal	ENCFF770UYJ	bed	methylation	hg38	Whole genome bisulfite-seq	paired	Illumina HiSeq 2000	Roadmap Epigenomics 2015
keratinocytes	primary cells	normal	GSM1127056	wig	methylation	hg19	Whole genome bisulfite-seq	paired	Illumina HiSeq 2000	Roadmap Epigenomics 2015
duodenum crypt	primary cells	normal	GSE141254	txt	methylation	not applicable	Infinium methylation assay	not applicable	HumanMethylation450 BeadChips Infinium MethylationEPIC arrays	Lewis et al. 2020
adipose	tissue	normal	ENCFF318AMC	bed	methylation	hg38	Whole genome bisulfite-seq	single	Illumina HiSeq 2500	Roadmap Epigenomics, 2015
esophagus	tissue	normal	ENCFF625GVK	bed	methylation	hg38	Whole genome bisulfite-seq	single	Illumina HiSeq 2500	Roadmap Epigenomics, 2015
heart (left ventricule)	tissue	normal	ENCFF536RSX	bed	methylation	hg38	Whole genome bisulfite-seq	single	Illumina HiSeq 2500	Roadmap Epigenomics, 2015
liver	tissue	normal	ENCFF356KGQ	bam	methylation	not applicable	Whole genome bisulfite-seq	single	Illumina HiSeq 2500	Roadmap Epigenomics, 2015
lung	tissue	normal	ENCFF039JFT	bed	methylation	hg38	Whole genome bisulfite-seq	single	Illumina HiSeq 2500	Roadmap Epigenomics, 2015
pancreas	tissue	normal	ENCFF763RUE	bed	methylation	hg38	Whole genome bisulfite-seq	single	Illumina HiSeq 2500	Roadmap Epigenomics, 2015
pre-frontal cortex	tissue	normal	SRR3278486_SRR3278482	FastQ	methylation	not applicable	Whole genome bisulfite-seq	paired	Illumina HiSeq 2500	Jenkinson et al. 2017 (PMID: 28346445)
sigmoid colon	tissue	normal	ENCFF157POM	bed	methylation	hg38	Whole genome bisulfite-seq	single	Illumina HiSeq 2500	Roadmap Epigenomics, 2015
skin (lower leg)	tissue	normal	ENCFF219GCQ	bed	methylation	hg38	Whole genome bisulfite-seq	paired	Illumina HiSeq X Ten	ENCODE Project Consortium, 2012
small intestine	tissue	normal	ENCFF241AQC	bed	methylation	hg38	Whole genome bisulfite-seq	single	Illumina HiSeq 2500	Roadmap Epigenomics, 2015
stomach	tissue	normal	ENCFF497YOO	bed	methylation	hg38	Whole genome bisulfite-seq	single	Illumina HiSeq 2500	Roadmap Epigenomics, 2015
testis	tissue	normal	ENCFF715DMX	bed	methylation	hg38	Whole genome bisulfite-seq	paired	Illumina HiSeq X Ten	ENCODE Project Consortium, 2012
thyroid	tissue	normal	ENCFF223LJW	bed	methylation	hg38	Whole genome bisulfite-seq	paired	Illumina HiSeq X Ten	ENCODE Project Consortium, 2012
alveolar type II	primary cells	normal	SRR1852851	FastQ	expression	not applicable	bulk RNA-seq	paired	Illumina HiSeq 2000	Marconett et al. 2017
adipose	tissue	normal	ERR315332_ERR315431 ERR315343_ERR315342 ERR315378	FastQ	expression	not applicable	bulk RNA-seq	paired	Illumina HiSeq 2000	Fagerberg et al. 2014 (PMID: 24309898)
colon	tissue	normal	ERR315357_ERR315484 ERR315462_ERR315400 ERR315348_ERR315403	FastQ	expression	not applicable	bulk RNA-seq	paired	Illumina HiSeq 2000	Fagerberg et al. 2014
cerebral cortex	tissue	normal	ERR315455_ERR315432 ERR315477	FastQ	expression	not applicable	bulk RNA-seq	paired	Illumina HiSeq 2000	Fagerberg et al. 2014

esophagus	tissue	normal	ERR315411_ERR315398 ERR315489_ERR315434 ERR315472_ERR315362	FastQ	expression	not applicable	bulk RNA-seq	paired	Illumina HiSeq 2000	Fagerberg et al. 2014
heart	tissue	normal	ERR315384_ERR315328 ERR315356_ERR315367 ERR315413_ERR315435 ERR315389_ERR315331 ERR315430	FastQ	expression	not applicable	bulk RNA-seq	paired	Illumina HiSeq 2000	Fagerberg et al. 2014
small intestine	tissue	normal	ERR315344_ERR315381 ERR315408_ERR315409 ERR315423_ERR315388 ERR315419_ERR315364	FastQ	expression	not applicable	bulk RNA-seq	paired	Illumina HiSeq 2000	Fagerberg et al. 2014
liver	tissue	normal	ERR315327_ERR315414 ERR315463_ERR315394 ERR315451	FastQ	expression	not applicable	bulk RNA-seq	paired	Illumina HiSeq 2000	Fagerberg et al. 2014
lung	tissue	normal	SRR577579_SRR577582	FastQ	expression	not applicable	bulk RNA-seq		Illumina HiSeq 2000	Roadmap Epigenomics, 2015
pancreas	tissue	normal	ERR315466_ERR315436 ERR315479_ERR315429	FastQ	expression	not applicable	bulk RNA-seq	paired	Illumina HiSeq 2000	Fagerberg et al. 2014
skin	tissue	normal	ERR315339_ERR315401 ERR315460_ERR315376 ERR315372_ERR315464	FastQ	expression	not applicable	bulk RNA-seq	paired	Illumina HiSeq 2000	Fagerberg et al. 2014
stomach	tissue	normal	ERR315379_ERR315369 ERR315467_ERR315485	FastQ	expression	not applicable	bulk RNA-seq	paired	Illumina HiSeq 2000	Fagerberg et al. 2014
testis	tissue	normal	ERR315352_ERR315415 ERR315492	FastQ	expression	not applicable	bulk RNA-seq	paired	Illumina HiSeq 2000	Fagerberg et al. 2014
thyroid	tissue	normal	ERR315363_ERR315397 ERR315358_ERR315412 ERR315428_ERR315491 ERR315483_ERR315422 ERR315337	FastQ	expression	not applicable	bulk RNA-seq	paired	Illumina HiSeq 2000	Fagerberg et al. 2014

Table S4. Transcriptomic datasets of cell lines treated with 5-azadC.

Sample	Type	Pathology	Name	Accession	File	Assay	Method	Layout	Platform	Reference
MCF7	cell line	tumor	MCF7_RNAseq_control_1day-Normoxia_r1a	SRR7822252	FastQ	expression	bulk RNA-seq	single	Illumina HiSeq 2500	D'Anna et al. 2020
MCF7	cell line	tumor	MCF7_RNAseq_control_1day-Normoxia_r1b	SRR7822253	FastQ	expression	bulk RNA-seq	single	Illumina HiSeq 2500	D'Anna et al. 2020
MCF7	cell line	tumor	MCF7_RNAseq_control_1day-Normoxia_r2a	SRR7822254	FastQ	expression	bulk RNA-seq	single	Illumina HiSeq 2500	D'Anna et al. 2020
MCF7	cell line	tumor	MCF7_RNAseq_control_1day-Normoxia_r2b	SRR7822255	FastQ	expression	bulk RNA-seq	single	Illumina HiSeq 2500	D'Anna et al. 2020
MCF7	cell line	tumor	MCF7_RNAseq_control_1day-Normoxia_r3a	SRR7822256	FastQ	expression	bulk RNA-seq	single	Illumina HiSeq 2500	D'Anna et al. 2020
MCF7	cell line	tumor	MCF7_RNAseq_control_1day-Normoxia_r3b	SRR7822257	FastQ	expression	bulk RNA-seq	single	Illumina HiSeq 2500	D'Anna et al. 2020
MCF7	cell line	tumor	MCF7_RNAseq_aza_1day-Normoxia_r1a	SRR7822264	FastQ	expression	bulk RNA-seq	single	Illumina HiSeq 2500	D'Anna et al. 2020
MCF7	cell line	tumor	MCF7_RNAseq_aza_1day-Normoxia_r1b	SRR7822265	FastQ	expression	bulk RNA-seq	single	Illumina HiSeq 2500	D'Anna et al. 2020
MCF7	cell line	tumor	MCF7_RNAseq_aza_1day-Normoxia_r2a	SRR7822266	FastQ	expression	bulk RNA-seq	single	Illumina HiSeq 2500	D'Anna et al. 2020
MCF7	cell line	tumor	MCF7_RNAseq_aza_1day-Normoxia_r2b	SRR7822267	FastQ	expression	bulk RNA-seq	single	Illumina HiSeq 2500	D'Anna et al. 2020
MCF7	cell line	tumor	MCF7_RNAseq_aza_1day-Normoxia_r3a	SRR7822268	FastQ	expression	bulk RNA-seq	single	Illumina HiSeq 2500	D'Anna et al. 2020
MCF7	cell line	tumor	MCF7_RNAseq_aza_1day-Normoxia_r3b	SRR7822269	FastQ	expression	bulk RNA-seq	single	Illumina HiSeq 2500	D'Anna et al. 2020
TS603	cell line	tumor	TS603_DMSO_rep1	SRR12105780	FastQ	expression	bulk RNA-seq	single	Illumina HiSeq 2000	Park et al. 2021
TS603	cell line	tumor	TS603_DMSO_rep2	SRR12105781	FastQ	expression	bulk RNA-seq	single	Illumina HiSeq 2000	Park et al. 2021
TS603	cell line	tumor	TS603_DAC_rep1	SRR12105782	FastQ	expression	bulk RNA-seq	single	Illumina HiSeq 2000	Park et al. 2021
TS603	cell line	tumor	TS603_DAC_rep2	SRR12105783	FastQ	expression	bulk RNA-seq	single	Illumina HiSeq 2000	Park et al. 2021
HMLER	cell line	normal immortalized	HMLER_DAC_1	SRR3362409	FastQ	expression	bulk RNA-seq	single	Illumina HiSeq 2000	Grandin et al. 2016
HMLER	cell line	normal immortalized	HMLER_DAC_2	SRR3362410	FastQ	expression	bulk RNA-seq	single	Illumina HiSeq 2000	Grandin et al. 2016
HMLER	cell line	normal immortalized	HMLER_Ctrl1	SRR3362411	FastQ	expression	bulk RNA-seq	single	Illumina HiSeq 2000	Grandin et al. 2016
HMLER	cell line	normal immortalized	HMLER_Ctrl2	SRR3362412	FastQ	expression	bulk RNA-seq	single	Illumina HiSeq 2000	Grandin et al. 2016

Table S5. Antibodies and tissue section codes from HPA.

Protein	DDIC	Antibody	Tissue	Pathology	Gender	Age	Patient_ID
MUC13	GI	HPA045163	Lung	Normal	Male	65	1470
VIL1	GI	HPA006884	Lung	Normal	Female	49	2268
A2ML1	SE	HPA038847	Esophagus	Normal	Female	66	3399
A2ML1	SE	HPA038847	Skin	Normal	Male	52	3338
A2ML1	SE	HPA038847	Vagina	Normal	Female	40	2276
A2ML1	SE	HPA038847	Lung	Normal	Male	65	1470
SERPINB5	SE	CAB009570	Esophagus	Normal	Male	54	3197
SERPINB5	SE	CAB009570	Skin	Normal	Male	16	2549
SERPINB5	SE	CAB009570	Vagina	Normal	Female	44	2480
SERPINB5	SE	CAB009570	Lung	Normal	Female	49	2268
MUC13	GI	HPA045163	LUAD	Tumor	Female	69	2777
VIL1	GI	HPA006884	LUAD	Tumor	Female	70	3391
A2ML1	SE	HPA038847	LUAD	Tumor	Female	76	448
SERPINB5	SE	CAB009570	LUAD	Tumor	Female	70	3391

Table S6. siRNAs used for transfection experiments.

siRNA	Sequence (5'-3')	Reference	Manufacturer
siHNF4A	GAC-CGG-AUC-AGC-ACU-CGA-A<br>CGG-AAG-AAC-CAC-AUG-UAC-U<br>GGG-CUG-GCA-UGA-AGA-AGG-A<br>CCA-AGU-ACA-UCC-CAG-CUU-U	L-003406-00-0020<br>ON-TARGETplus SMARTpool	Dharmacon
siLuciferase	CUUACGCUGAGUACUUCGA	Tilman et al. 2012	Eurogentec

Table S7. Primer sequences, PCR anq qPCR reagents and conditions.

Gene	Forward primer (5'-3')	Reverse primer (5'-3')	Assay	Kit	Manufacturer	Tempature/Time	Cycles
NAP1L1	CCT-GGA-TCT-GAG-AGC-TTC-TCT-T	ACA-CCG-CTC-GCG-ATC-CAA-T	PCR	DreamTaq	ThermoFischer	Annealing: 60°C, 30s<br>Extension: 72°C, 30s	36
NAA11	GCA-GTG-ACA-GCA-AAG-AAC-CTA	GAT-CCC-AGC-AGG-ATA-TGT-GAA	PCR	DreamTaq	ThermoFischer	Annealing: 60°C, 30s<br>Extension: 72°C, 30s	36
EPS8L3	TGA-GCT-CGT-ACA-CAT-CCT-CTT	ACA-GGG-TCC-TGG-TAT-CCT-A	PCR	DreamTaq	ThermoFischer	Annealing: 60°C, 30s<br>Extension: 72°C, 30s	36
VIL1	CGT-GTT-CAA-TGC-TAA-CAG-CAA-C	ATG-AGA-CCC-TAC-AAT-CAG-GGT-A	PCR	DreamTaq	ThermoFischer	Annealing: 60°C, 30s<br>Extension: 72°C, 30s	36
GJB5	GCA-GGC-TCT-GTC-CTG-GAA-ACA	CGA-GTA-TTG-CAG-TCG-AAG-TCC-T	PCR	DreamTaq	ThermoFischer	Annealing: 60°C, 30s<br>Extension: 72°C, 30s	36
SERPINB5	TTC-CAG-GAT-AAC-TGT-GAC-T	TCC-AAA-GGG-TAC-ATC-TTT-GAC-A	PCR	DreamTaq	ThermoFischer	Annealing: 60°C, 30s<br>Extension: 72°C, 30s	36
MAGEA1	GCC-GAA-GGA-ACC-TGA-CC	ACT-GGG-TTG-CCT-CTG-TCG	PCR	DreamTaq	ThermoFischer	Annealing: 62°C, 30s<br>Extension: 72°C, 1min	35
PGLYRP3	CGT-CTA-CAC-CAT-AGG-CTG-GT	CCT-TCT-GGA-TGG-CAT-AGG-AGA-TCA	PCR	DreamTaq	ThermoFischer	Annealing: 60°C, 30s<br>Extension: 72°C, 30s	36
ACTB	CCC-TGG-ACT-TCG-AGC-AAG-AGA-T	AAG-GTA-GTT-TCG-TGG-ATG-CCA-CA	PCR	DreamTaq	ThermoFischer	Annealing: 60°C, 30s<br>Extension: 72°C, 30s	20
HNF4A	CAT-ACG-CAT-CCT-TGA-CGA-GCT	GAT-GAA-CTG-GAT-CTG-CTC-GAT	qPCR	KAPA SYBR FAST	Sigma Aldrich	Annealing: 60°C, 30s<br>Extension: 60°C, 30s	40
MUC13	GCC-ATC-ATT-CAT-CTT-ACT-CTT-CT	TCA-CTG-TCT-GCA-GCA-GTA-GGT	qPCR	KAPA SYBR FAST	Sigma Aldrich	Annealing: 60°C, 30s<br>Extension: 60°C, 30s	40
CTGABRA3	GAA-AGA-AAG-AAA-GGT-CAC-AGG-TCT-CT	GCC-AAT-GTC-CTG-CTT-CAC-AAA-GT	qPCR	KAPA SYBR FAST	Sigma Aldrich	Annealing: 60°C, 30s<br>Extension: 60°C, 30s	40
ACTB	CCC-TGG-ACT-TCG-AGC-AAG-AGA-T	AAG-GTA-GTT-TCG-TGG-ATG-CCA-CA	qPCR	KAPA SYBR FAST	Sigma Aldrich	Annealing: 60°C, 30s<br>Extension: 60°C, 30s	40
EPS8L3	TGA-GCT-CGT-ACA-CAT-CCT-CTT	ACA-GGG-TCC-TGG-TAT-CCT-A	qPCR	KAPA SYBR FAST	Sigma Aldrich	Annealing: 60°C, 30s<br>Extension: 60°C, 30s	40
VIL1	CGT-GTT-CAA-TGC-TAA-CAG-CAA-C	ATG-AGA-CCC-TAC-AAT-CAG-GGT-A	qPCR	KAPA SYBR FAST	Sigma Aldrich	Annealing: 60°C, 30s<br>Extension: 60°C, 30s	40
